# Enriching microbes capable of fluorotelomer acid defluorination: thermodynamic constraints and experimental challenges

**DOI:** 10.1038/s44454-026-00034-4

**Published:** 2026-06-01

**Authors:** Dan Wang, Bei Yan, Nancy N. Perreault, Jinxia Liu

**Affiliations:** 1 Department of Civil Engineering, McGill University, Montreal, QC Canada; 2 National Research Council of Canada, Montreal, QC Canada; 3 Department of Civil and Environmental Engineering, Hong Kong Polytechnic University, Hung Hom, Hong Kong SAR

**Keywords:** Biotechnology, Environmental sciences, Microbiology

## Abstract

Fluorotelomer compounds are a major class of polyfluoroalkyl substances that can undergo partial microbial transformation via the one‑carbon removal pathway, which has the potential to achieve deep defluorination. However, the microorganisms and mechanisms driving this pathway remain elusive. We focus on 5:3 fluorotelomer carboxylic acid (5:3 FTCA) because, although its structure suggests susceptibility to biodegradation, it exhibits high environmental persistence, making it a useful model for probing the feasibility and constraints of the one‑carbon removal pathway. In this study, we combine density functional theory (DFT)‑based thermodynamic modeling with extensive experimental screening to evaluate the feasibility of enriching microorganisms capable of n:3 fluorotelomer acid (n:3 FTCA) defluorination. DFT calculations reveal that the overall pathway is energetically favorable under alkaline conditions, with dehydrogenation and hydroxylation, rather than defluorination, likely serving as rate‑limiting steps. Despite extensive screening of activated sludge and soils, pure bacterial cultures, and extreme-pH-tolerant enriched consortia, no individual degraders were identified, indicating that such microbes are rare and likely dependent on cooperative community interactions. Limited biotransformation of 5:3 FTCA to 4:3 FTCA was observed in mixed consortia containing a methylotroph, confirming the co‑metabolic nature of this process and demonstrating the limitations of high‑throughput assays for cultivating kinetically constrained degraders. To address these challenges, we propose a dual-stage enrichment strategy decoupling survival from functional selection: initial maintenance followed by targeted co-metabolic enrichment. Overall, this study clarifies the energetic basis and enrichment constraints of fluorotelomer degradation and provides a framework for cultivating microbes capable of the one-carbon removal pathway.

## Introduction

Per- and polyfluoroalkylsubstances (PFAS) are a large class of synthetic chemicals used globally in industrial and consumer products for their surfactant properties, chemical stability, and resistance to heat and degradation^1,2^. Their environmental release and extreme persistence pose significant human and ecological risks. Among PFAS, perfluoroalkyl carboxylates (PFCAs) and sulfonates (PFSAs) are particularly notable for their global distribution and recalcitrance, having been detected in nearly all environmental compartments^3,4^. These concerns are compounded by their bioaccumulation and toxicity^5,6^; for instance, perfluorooctanoic acid (PFOA) is classified as possibly carcinogenic to humans by the International Agency for Research on Cancer^7^.

**Fig. 1:**
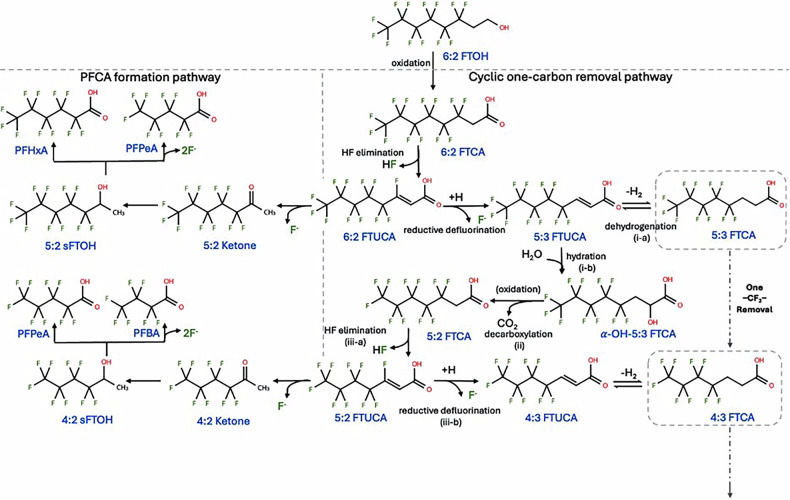
Proposed biotransformation pathway of fluorotelomer compounds leading to PFCA formation or one-carbon removal. Biotransformation pathway 6:2 FTOH to generate 5:3 and 4:3 FTCAs, showing two major downstream routes (i) the PFCA pathway and (ii) the one‑carbon removal pathway, based on the experimental observations reported by Wang et al.^18^. Arrows indicate individual biotransformation steps.

Understanding PFAS transformation is critical for risk assessment and remediation. Despite reports of PFSA or PFCA biotransformation, clear evidence for microbial degradation of these perfluoroalkyl compounds remains weak and inconsistent^8,9^. Therefore, the current scientific consensus remains that these *perfluoroalkyl* compounds do not biodegrade under environmentally relevant conditions. In contrast, *polyfluoroalkyl* compounds such as n:2 fluorotelomer alcohols (n:2 FTOHs) and their derivatives have been frequently reported to biotransform in activated sludge, soil, and microbial or fungal cultures^10–16^. Biotransformation of n:2 FTOHs proceeds via two competing pathways with different outcomes (Fig. 1). The “PFCA formation pathway” produces dead-end PFCAs, such as PFOA, PFHxA, and PFBA^13,17,18^, which are common but undesirable outcomes. The “one-carbon removal pathway”, in contrast, generates n:3 (C_n_F_2n+1_CH_2_CH_2_CO_2_H) fluorotelomer acids (n:3 FTCAs) and other fluorinated intermediates and proceeds via stepwise carbon elimination, cleaving off one carbon and removing two fluorine atoms per cycle. In theory, repetition of this process could shorten and potentially mineralize the entire perfluoroalkyl chain, representing a fundamentally different and more complete degradation mechanism. However, this pathway remains underexplored^13,17,18^, and factors controlling substrate flux between the two interlinked pathways remain poorly understood. In many studies, 5:3 fluorotelomer acid (5:3 FTCA), a major transformation product of 6:2 FTOH, is surprisingly persistent despite containing three non‑fluorinated carbons.

Wang et al. used isotope-labeled 5:3 FTCA to confirm the feasibility of biotransformation via the one-carbon removal pathway, identifying multiple transformation intermediates but observing slow kinetics^18^. They proposed that bypassing acyl-CoA synthetase activation makes the conversion of 5:3 FTCA to 4:3 FTCA thermodynamically feasible. They further estimated that for each ─CF_2_─ removal cycle (e.g., from 5:3 FTCA to 4:3 FTCA) could generate up to 6 ATPs or 2 [NAD(P)H^+^] equivalents. This yield is comparable to octanoic acid ß-oxidation, which produces approximately 7.6 ATPs per carbon mineralized^18^. Despite these findings, no n:3 FTCA-degrading microbes have yet been isolated, suggesting unresolved ecological, physiological, or methodological barriers.

Successful isolation of xenobiotic-degrading microorganisms requires both thermodynamic feasibility and appropriate enzymatic machinery. For n:3 FTCAs in particular, a bioenergetic assessment remains limited, making it difficult to identify which reactions are unfavorable and which are slow or biologically inaccessible. Understanding the Gibbs free energy (*ΔG*) changes for each step is crucial for identifying energetic bottlenecks and for formulating experimentally testable hypotheses about what limits pathway flux. Such an evaluation can be achieved using Density Functional Theory (DFT), which allows estimation of reaction energetics for proposed intermediates and transformations^19,20^. However, even thermodynamically favorable pathways may not be realized biologically. The strength of the C-F bond can make PFAS biodegradation both energetically and kinetically demanding^21,22^. In addition, the scarcity of naturally occurring organofluorines implies limited evolutionary selection, which may restrict the development of specialized defluorinating enzymes^23,24^. Even so, biological defluorination has been repeatedly observed in FTOHs and derivatives^25,26^, indicating that C-F bond strength alone cannot explain when transformation occurs. Another critical barrier is the limitation of enrichment techniques. Conventional methods typically rely on growth-coupled selection, providing the contaminant as the sole carbon source or an essential nutrient. This strategy has succeeded for 6:2 fluorotelomer sulfonate (6:2 FTSA) under sulfur-limiting conditions, where microorganisms (e.g., *Gordonia* sp. NB4-1Y) utilize sulfur from 6:2 FTSA^27,28^. However, this conventional approach may systematically miss co-metabolic, slow, or community-dependent transformations. Consistent with this, existing studies suggest that many fluorotelomers do not support growth directly^26^ but are instead transformed co-metabolically by nonspecific enzymes^29^. These observations motivate enrichment and screening strategies that prioritize transformation activity over growth on the target compound.

Therefore, this study investigates n:3 FTCA transformation from complementary theoretical and experimental perspectives. We evaluated the thermodynamic feasibility of the one-carbon removal pathway using DFT to calculate Δ*G*° for key reaction steps. In parallel, we conducted a comprehensive experimental search for 5:3-FTCA-transforming microorganisms, using high-throughput screening of soil and activated sludge, pure cultures, and defined consortia. Lastly, we identified bottlenecks that can hinder microbial enrichment of rare or community-dependent degraders and proposed a conceptual dual-stage strategy that decouples microbial survival from functional selection. By integrating theoretical and experimental insights, we aim to elucidate the viability of the one‑carbon removal pathway and establish a logically derived framework to guide future discovery and cultivation of fluorotelomer-degrading microbial consortia.

## Results

### DFT-based thermodynamic analysis

**Fig. 2:**
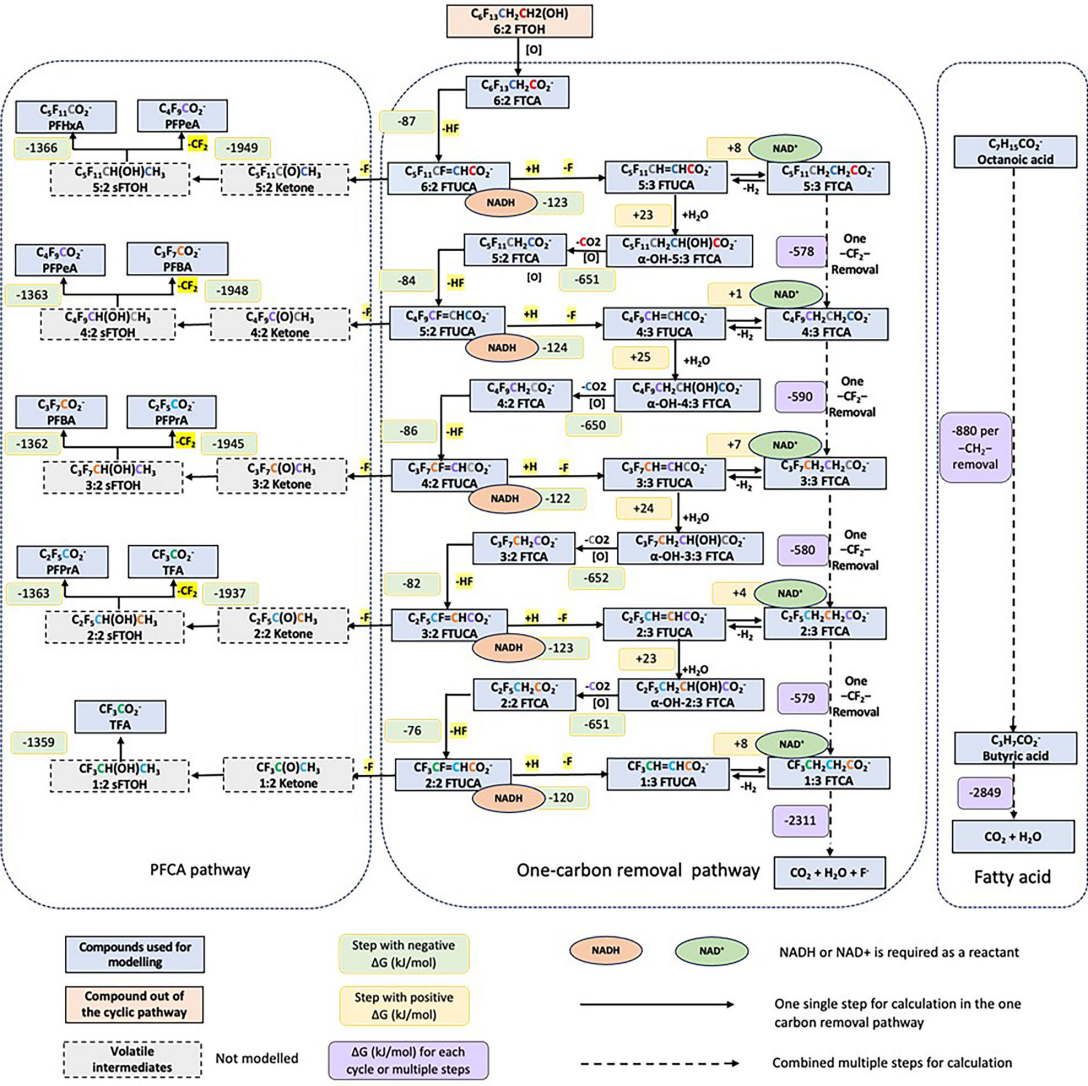
Thermodynamic profiles of fluorotelomer acid biotransformation pathways. Calculated Gibbs free energy changes for individual reaction steps in fluorotelomer acid biotransformation via the PFCA formation pathway and the one-carbon removal pathway, with comparison to hydrocarbon fatty acid oxidation. Negative values indicate exergonic reactions and positivee values indicate energetically unfavourable steps; calculation equations are provided in Supplementary Table S3.

Our thermodynamic modeling aimed to determine which degradation steps are energetically favorable and which may be rate-limiting for n:3 FTCA transformation. Three related but distinct concepts are relevant: (1) *thermodynamic favorability*, whether a reaction can occur spontaneously based on Gibbs free energy; (2) *biological accessibility*, whether organisms possess the enzymatic machinery to catalyze the reaction; and (3) *kinetic feasibility*, how fast the reaction proceeds under biological conditions. A thermodynamically favorable reaction may still be biologically inaccessible if appropriate enzymes are absent, or kinetically slow if the activation energy high. Overall, our calculations (Fig. 2) indicate that the one-carbon removal pathway is thermodynamically feasible under alkaline conditions, yet its biological realization is evidently rare, suggesting that kinetic or enzymatic constraints, rather than thermodynamic limitations, represent the primary barriers to n:3 FTCA transformation. For the thermodynamic analysis, we first established the role of pH in reaction energetics, and then compared the Gibbs free energy changes between the one-carbon removal pathway and the PFCA formation pathways to identify potential reaction bottlenecks.

The critical role of pH became apparent when balancing the reactions using common reactants such as H_2_O, CO_2_, H^+^, and OH^−^. Specifically, it was observed that during the transformation from n:3 FTCA to (n-1):3 FTCA or n:3 FTUCA to (n-1):3 FTUCA, two H^+^ ions are released per CO_2_ molecule generated. The release of H^+^ shifts Δ*G*° at different pH values. The entire n:3 FTCA degradation pathway is thermodynamically feasible when OH^−^ is the reactant rather than H_2_O, indicating that the process is favored under alkaline conditions, as illustrated below for 5:3 FTCA at 298.15 K and 1.00 atm.

**Example i**: 5:3 FTCA degradation to 4:3 FTCA (loss of 1C and 2F) E

1. 5:3 FTCA^−^ + 0.5 O_2_ + H_2_O → 4:3 FTCA^−^ + 2 H^+^ + 2 F^−^ + CO_2_ (at pH < 7: Δ*G*° = +780 kJ/mol)
2. 5:3 FTCA^−^ + 0.5 O_2_ + 2 OH^−^ → 4:3 FTCA^−^ + H_2_O + 2 F^−^ + CO_2_ (at pH > 7: Δ*G*° = −578 kJ/mol)
  **Examples ii**: 5:3 FTCA degradation to PFPeA (loss of 2 C and 2 F)
3. 5:3 FTCA^−^ + 3.5 O_2_ → PFPeA^−^ + 3 CO_2_ + 2 F^−^ + 2 H^+^ + H_2_O (at pH < 7: Δ*G*° = +225 kJ/mol)
4. 5:3 FTCA^−^ + 3.5 O_2_ + 2 OH^−^ → PFPeA^−^ + 3 CO_2_ + 2 F^−^ + 3 H_2_O (at pH > 7: Δ*G*° = −2317 kJ/mol)
  **Examples iii**: 5:3 FTCA degradation to PFBA (loss of 4C and 4F)
5. 5:3 FTCA^−^ + 4 O_2_ → PFBA^−^ + 4 CO_2_ + 4 F^−^ + 4 H^+^ (at pH < 7: Δ*G*° = −183 kJ/mol)
6. 5:3 FTCA^−^ + 4 O_2_ + 4 OH^−^ → PFBA^−^ + 4 CO_2_ + 4 F^−^ + 4 H_2_O (at pH > 7: Δ*G*° = −2901 kJ/mol)

The n:3 FTCA formation pathway (Example i) involves the loss of one −CF_2_− to form (n-1):3 FTCA and the eventual release of F^−^, CO_2_ and H^+^. This step is thermodynamically feasible only under alkaline conditions or when the produced H^+^ is readily consumed. In contrast, the PFCA formation pathways (loss of one or two −CF_2_− to form corresponding PFCAs) are more favorable under alkaline conditions (Example ii) or under both acid and alkaline conditions (Example iii). This might explain why the PFCA formation pathway is more commonly observed in the environment. Accordingly, only the reactions under alkaline conditions were used to calculate Δ*G* for the n:3 FTCA pathway (Fig. 2); additional details are provided in Supplementary Tables S2, S3.

The energy yield of the one-carbon removal pathway, the PFCA pathway, and the fatty acid oxidation pathway was compared in Fig. 2. For the purpose of energy calculations (Fig. 2), pathways are considered to begin at 6:2 FTCA, proceed to 5:3 FTCA, then stepwise to 1:3 FTCA, followed by its ultimate mineralization. The degradation steps from 5:3 FTCA to 3:3 FTCA have been experimentally observed^18,27^, but the subsequent steps from 3:3 FTCA to 1:3 FTCA remain hypothetical and are proposed based on analogous reaction mechanisms, differing only in the length of the perfluoroalkyl carbon chain. As shown in Fig. 2, along the degradation pathway from 5:3 FTCA to 1:3 FTCA, a total of 2327 kJ/mol of energy is released, corresponding to 582 kJ/mol per carbon removed, or the equivalent of 8.3 ATP equivalents. Synthesizing 1 mol of ATP requires ~70 kJ/mol under conditions in actively growing cells, accounting for heat loss^30^. For comparison, hydrocarbon fatty acid oxidation and the PFCA formation pathways were also calculated. In the oxidation of octanoic acid to butyric acid (Octanoic acid + 6 O_2_ → Butyric acid- + 4 CO_2_ + 4 H_2_O; pH > 7, Δ*G°* = −3519 kJ/mol), the removal of each carbon releases 880 kJ/mol on average, or the equivalent of 12.6 ATP per carbon. The PFCA formation pathway and the n:3 FTCA pathway are interconnected, with energy released from n:2 FTUCAs shortening to the corresponding PFCA being similar across different chain lengths. For example, conversion of 5:3 FTCA to PFPeA (Example ii) releases 2317 kJ when O_2_ is the sole electron acceptor (Equation 2), which means that the removal of each carbon releases 790 kJ/mol on average, or the equivalent of 11.3 ATP per carbon. Similarly, the conversion of 5:3 FTCA to PFBA (Example iii) releases 2901 kJ, or 10.4 ATP equivalents per carbon. Assuming equal formation of PFPeA and PFBA, the PFCA route averages 10.9 ATP equivalents per carbon removed.

Overall, the one‑carbon removal pathway releases ~66% of the energy produced during typical hydrocarbon fatty acid mineralization, while the PFCA formation pathway releases ~86% of that energy. However, because the PFCA route ends in stable, nondegradable PFCA, the one‑carbon removal pathway, in theory, could ultimately produce more energy by allowing continued carbon chain shortening. If 1:3 FTCA is mineralized to fully defluorinated products, an additional 2311 kJ/mol of energy could be released; this route averages 578 kJ/mol (8.3 ATP equivalent) per carbon, compared with 712 kJ/mol (10.2 ATP equivalent) per carbon for non-fluorinated butyric acid. Together, these calculations suggest that the n:3 FTCA pathway may enable microbes to target the persistent fluorinated backbone to extract the remaining chemical energy.

However, several uncertainties affect these calculations, including the identities of reactants and products, intermediates, pH conditions, and the enzymatic complexities. The simplified calculations used here may not fully capture enzymatic mechanisms. Nevertheless, the calculations show that the one-carbon removal pathway is thermodynamically feasible. A previous study estimated that removing one CF_2_ from 5:3 FTCA to form 4:3 FTCA may produce up to 6 ATP equivalents or 2 [NAD(P)H + H+]^18^. Our calculated energy change of −578 kJ/mol (≈8.3 ATP equivalents) is broadly consistent with that estimate. In addition, although standard Δ*G*° values are useful for comparing relative exergonicity between steps, they likely overestimate the spontaneity of fluorotelomer defluorination in real environments where compound concentrations are low.

### Identification of the energetic bottleneck

Thermodynamically constrained reactions may create key bottlenecks in the n:3 FTCA transformation pathway. Based on Fig. 2, two energetically challenging steps were identified: (a) dehydrogenation of n:3 FTCA to n:3 FTUCA with NADH produced and (b) hydroxylation of the n:3 FTUCA to α-OH-5:3 FTCA. For (a) dehydrogenation of n:3 FTCA → n:3 FTUCA, it is typically catalyzed by dehydrogenase enzymes. When coupled to the NAD⁺/NADH system, the calculated Δ*G* is near zero for 5:3 FTCA or shorter analogs (±1–8 kJ mol⁻¹, Fig. 2), suggesting near thermodynamic equilibrium and reversibility. Such small ΔG differences imply that the reaction proceeds efficiently only when the product (n:3 FTUCA) is rapidly consumed or when it is coupled to a strongly exergonic reaction. The direction in which it proceeds is highly dependent on the intracellular concentrations of NAD⁺ and NADH. When O₂ is the electron acceptor, however, the reaction becomes strongly exergonic (Supplementary Table S3, reactions 15–19), providing a stronger driving force under aerobic conditions. With NAD⁺ as the electron acceptor, the energy is largely conserved in NADH, which can supply reducing power for subsequent reductive defluorination. Ultimately, whether the dehydrogenation is truly rate‑limiting depends on how quickly n:3 FTUCA is converted to α‑OH n:3 FTCA. Experimental work with activated sludge previously suggested that 5:3 FTCA → 5:3 FTUCA is rate‑limiting^18^.

For (b) hydroxylation of n:3 FTUCA→α-OH n:3 FTCA, the step consistently shows a positive Δ*G* (23–25 kJ mol⁻¹; Fig. 2), indicating that it requires energy input and may be the true rate‑limiting step. Because the same hydroxylation is required at each round of one‑carbon removal, this energetic barrier repeats in every cycle. In general, hydroxylation requires reducing equivalents (e.g., NAD(P)H) and an oxidant (e.g., O_2_) and is often catalyzed by monooxygenase or cytochrome P450 enzymes, which insert a hydroxyl group into an unactivated C-H or C-F site; hydroxylation adjacent to fluorinated carbons can further increase the energetic and enzymatic demands, and these reactions increase the energeic and enzymatic demands^31^. For n:3 FTUCA, α‑hydroxylation occurs adjacent to a carboxylate, which requires activating a stable carbon center and is further disfavored by electron‑withdrawing effects of fluorine atoms. Experimentally, α‑OH intermediates (i.e., α‑OH-n:3 FTCA) are rarely detected, which suggests either low formation rates and/or rapid turnover once formed. In activated‑sludge experiments, α‑OH 5:3 FTCA was rapidly converted to 4:3 FTCA, about 76% within 2 days^18^. This suggests slow formation but fast consumption: once α‑OH-n:3 FTCA forms, it is readily decarboxylated or transformed downstream. The calculations likewise confirm that α-OH-n:3 FTCA **→** n:2 acid is strongly exergonic, which pulls the preceding unfavorable hydroxylation forward by rapidly consuming its product.

In comparison, the two defluorination steps in each cycle are unlikely to be rate-limiting. In the first defluorination reaction, HF is eliminated from n:2 FTCA to form n:2 FTUCA, releasing ~−76~−87 kJ mol⁻¹ and producing a partially hydrogenated intermediate. A second F elimination from n:2 FTUCA releases more energy (−120~123 kJ mol⁻¹), yielding n:3 FTUCA that then enters subsequent redox-coupled reactions. Although the second step (n:2 FTUCA → n:3 FTUCA) proceeds via reductive defluorination and therefore requires cellular reducing equivalents (e.g., NADH-linked electron transfer), the overall energetics remain strongly favorable. Together, these two exergonic steps contribute ~−200 kJ mol⁻¹, so they are not expected to constitute the primary thermodynamic bottleneck, provided sufficient reducing power is available. These HF elimination events appear essential: they generate the n:3 FTUCA required for downstream hydration and chain shortening, preventing the cycle from stalling at the n:2 FTCA stage. However, n:2 FTUCA is a key branch point, because it can also enter the PFCA formation pathway, yielding dead-end PFCAs.

The Δ*G°* values reported here (under standard conditions) are valuable for comparing relative exergonicity of each step, but in real environmental settings where PFAS concentrations are typically low, the actual Δ*G* becomes less negative (or even positive), potentially making even nominally exergonic reactions less favorable. In addition, reaction rates depend strongly on enzyme activity and regulation, as well as microbial metabolism, rather than on thermodynamics alone. Still, experimental evidence supports the feasibility of reductive defluorination even under aerobic conditions. For instance, pure cultures have been found to convert 6:2 FTUCA to 5:3 FTCA during 6:2 FTOH degradation^32–34^. Taken together, these results point to dehydrogenation and hydroxylation, rather than defluorination, as likely the most energetically and kinetically demanding steps. Because microbial activity can vary with community composition and other stochastic factors, screening diverse environmental samples is needed to evaluate how general and environmentally feasible these steps are. These putative bottlenecks also provide a framework for interpreting the experimental results below. If they require energy input or rare enzymes, we expect: (1) few microorganisms capable of performing the complete pathway; (2) transformation occurring preferentially in consortia where syntrophic interactions can provide necessary energetic coupling; and (3) partial transformation (e.g., hydroxylated intermediates) observed more frequently than complete one-carbon removal. As described in the following sections, our experimental observations are consistent with these predictions.

### High-throughput screening of environmental microbes

In the first stage of the experiment, we used dilution‑to‑extinction in a 96‑well plate to enrich metabolically active microorganisms that might transform FTCAs and to generate a library of microbial consortia. PFAS-contaminated soil and activated-sludge inocula were serially diluted to 10⁻⁶ in plates containing 10 ppm 5:3 FTCA and resazurin; wells turning pink after 7 days were scored as active. Because resazurin reports general metabolic activity rather than defluorination, this step served to identify viable wells for follow-up PFAS profiling. These metabolically active wells were tested for the production of 4:3 FTCA from 5:3 FTCA by LC-MS/MS. Initial screenings revealed little to no conversion of 5:3 FTCA, with only sporadic trace 4:3 FTCA near the detection limit. These wells were sub-cultured in new 96-well plates, but no measurable degradation was detected after 3 weeks. Unexpectedly, plates sealed with Parafilm and stored at 4 °C for ~3 months developed prominent biofilms and red flocs in several wells inoculated with activated sludge, even in the absence of added co-substrates. LC-MS/MS analysis revealed a significant 5:3 FTCA transformation, producing PFBA, PFPeA, and trace amounts of 4:3 FTCA (Table 1). Control wells remained blue, and other inocula showed no change. Because PFHxA was also present in controls, it was partly attributed to impurities and was not considered a definitive indicator of the transformation of 5:3 FTCA. The extent of 5:3 FTCA degradation correlated strongly with the presence of red flocs (Supplementary Table S6), whereas 5:3 FTCA remained unchanged in sterile wells and in wells without biofilms.

**Table 1:** PFAS profile (unit: ng/mL) for four selected microbial consortia after 3 months of storage at 4 °C in each individual well

	Parent compound	Transformation products			
	5:3 FTCA	4:3 FTCA	PFHxA	PFPeA	PFBA
Control 1	1.27 ×10⁴	n.d.	1.24 ×10²	n.d.	n.d.
Control 2	1.37 ×10⁴	n.d.	1.58 ×10²	n.d.	n.d.
Live 1	5.43 ×10³	1.62 ×10¹	1.80 ×10²	1.37 ×10²	9.93 ×10²
Live 2	3.85 ×10³	1.79 ×10¹	3.65 ×10²	2.34 ×10²	1.77 ×10³
Live 3	1.31 ×10⁴	4.15 ×10¹	3.75 ×10²	2.75 ×10²	2.41 ×10³
Live 4	6.43 ×10³	n.d.	1.96 ×10²	1.59 ×10²	1.07 ×10³

Control wells showed no microbial activities with resazurin remaining blue.

The difficulty in enriching n:3 FTCA degraders can be partially explained through our thermodynamic analysis. The hydroxylation step (n:3 FTUCA → α-OH-n:3 FTCA) is endergonic and may be a kinetic barrier. This could explain why transformation is mainly observed in mixed consortia, where syntrophic interactions can provide additional driving force, analogous to interspecies hydrogen transfer in methanogenic degradation^30^. The thermodynamic calculations also suggest that defluorination is more favorable when the H⁺ released is efficiently removed or neutralized. However, this does not mean that simply raising the external pH will help, because cells generally maintain a near‑neutral intracellular pH even in alkaline media. Consistent with this, the alkalinophilic consortium (pH 10.3) produced α/β‑hydroxy‑5:3 FTCA but no downstream products, indicating that pH alone is insufficient and that downstream conversion likely requires specific enzymes and/or partner organisms that rapidly consume the hydroxylated intermediate.

To identify the microorganisms associated with 5:3 FTCA transformation in the positive wells, the contents of the positive wells were subcultured onto R2A and MSM agar plates, yielding seven distinct isolates (Supplementary Table S9). The most prominent strain was a pink-pigmented isolate (isolate 1), present in every positive well. BLASTn analysis of its 16S rRNA gene identified the organism as *Methylobacterium* sp. (Supplementary Table S9), a facultative methylotroph. The strain grew only on MSM + methanol (Supplementary Fig. S1, d-1 and d-3), indicating that trace residual methanol (used to dissolve 5:3 FTCA) supported *Methylobacterium* growth. Although the methanol was visually evaporated, trace amounts could have remained inside the sealed wells and been sufficient for growth. Follow‑up tests (Supplementary Tables S6, S7) confirmed that *Methylobacterium sp*. alone did not degrade 5:3 FTCA, but its consistent presence suggests it supports the transforming consortium. One plausible role is indirect facilitation: methanol‑driven growth could supply reducing power, exchange metabolites, and deplete O₂ locally, thereby creating conditions that favor cometabolic transformation by partner organisms. Future studies should explicitly test this methylotroph’s role (e.g., by adding or removing Methylobacterium, controlling methanol at trace levels, and using co‑culture experiments with isotope tracing) to determine whether it is a keystone facilitator or simply a marker of favorable conditions. The other six isolates were identified as members of the genera *Mycobacterium, Pseudomonas, Sphingomonas, Stenotrophomonas, Bosea, and Thermomonas* (Supplementary Table S9), and they grew only on R2A (yellow colonies in Supplementary Fig. S1 d-2 and d-4). These isolates belong to two distinct bacterial phyla: Proteobacteria (six of them) and Actinobacteria (Mycobacterium). This taxonomic diversity suggests that the enrichment selected for a consortium of metabolically distinct microorganisms, several of which are known for hydrophobic compound metabolism and xenobiotic transformation. Each isolate was also tested individually for its ability to biotransform 5:3 FTCA using the carbon substrates listed in Table 2, but none of the isolates showed detectable transformation activity; 4:3 FTCA and other PFAS were not observed, and fluoride was below detection limits.

**Table 2:** Screening results of pure cultures and defined consortia, including previously characterized strains and newly isolated strains from the 5:3 FTCA enrichment cultures in this study

No.	Name	Rationale of selection	Co-substrates	Incubation period	Metabolite production	Defluorination
1	*Gordonia* sp. NB4-1Y	6:2 FTSA degrader	Glucose, Acetate, Butyrate, 3-Methyl pentanoic Acid, 3-Methyl hexanoic Acid, Nutrient broth PD-MSM-YE media	14 d	None	No
2	*Rhodococcus* sp. JVH1	Likely a 6:2 FTSA degrader	Glucose, Acetate, Butyrate, 3-Methyl pentanoic Acid, 3-Methyl hexanoic Acid, Nutrient broth PD-MSM-YE media	14 d	None	No
3	Mycobacterium *austroafricanum* IFP 2012	Methyl tert-butyl ether (MTBE) degrader	Octane	21 d	None	No
4	*Pseudomonas oleovorans*	Alkane degrader	Octane	21 d	None	No
5	Isolate 1 *Methylobacterium* sp.	Isolated in this study with 5:3 enrichment	Methanol, Octane, R2A medium	14 d	None	No
6	Isolate 2 *Sphingomonas* sp.	Isolated in this study with 5:3 enrichment	Methanol, Octane, R2A medium	14 d	None	No
7	Isolate 3 *Stenotrophomonas* sp.	Isolated in this study with 5:3 enrichment	Methanol, Octane, R2A medium	14 d	None	No
8	Isolate 4 *Bosea* sp.	Isolated in this study with 5:3 enrichment	Methanol, Octane, R2A medium	14 d	None	No
9	Isolate 5 *Mycobacterium* sp.	Isolated in this study with 5:3 enrichment	Methanol, Octane, R2A medium	14 d	None	No
10	Isolate 6 *Thermomonas* sp.	Isolated in this study with 5:3 enrichment	Methanol, Octane, R2A medium	14 d	None	No
11	Isolate 7 *Bosea* sp.	Isolated in this study with 5:3 enrichment	Methanol, Octane, R2A medium	14 d	None	No
12	Acid rock drainage consortium	Acid-tolerant consortium, pH = 2	NA	30 d	None	No
13	Alkaline consortium	Alkaline-tolerant consortium, pH = 10.3	NA	30 d	α/β‑hydroxy ‑5:3 FTCA	No

### Screening of additional pure cultures and defined consortia

To further evaluate 5:3 FTCA biotransformation potential, additional well‑characterized strains and defined consortia were examined, including four pure cultures (*Gordonia* sp.NB4-1Y, *Rhodococcus* sp.JVH1, *Mycobacterium austroafricanum* IFP 2012, and *Pseudomonas oleovorans*) and two defined microbial consortia (an acid‑tolerant consortium maintained at pH 2 and an alkali‑tolerant consortium maintained at pH 10.3). The strains were selected based on their ready availability and reported degradation capacities for PFAS, hydrocarbons, or methyl *tert*-butyl ether (MTBE). *Gordonia* sp.NB4‑1Y has been shown to transform FTSA and 6:2 fluorotelomer alkyl betaine (FTAB) under sulfur‑limiting conditions^27^. *Rhodococcus* sp. JVH1 has similar sulfur utilization traits, although fluorotelomer metabolism has not yet been evaluated^28^. *Mycobacterium austroafricanum* IFP 2012 can grow on MTBE as its sole carbon source^35^, and *P. oleovorans* degrades alkanes by expressing alkane hydroxylase^36^. For *Gordonia* and *Rhodococcus*, non-fluorinated carbon substrates (Table 2) were added individually to support activity, including 3‑methylpentanoic acid and 3‑methylhexanoic acid to induce α‑oxidation, which sequentially removes one carbon from branched fatty acids as CO₂, analogous to the one‑carbon removal pathway.

All cultures showed increases in biomass, confirming metabolic acidity, but none produced fluoride or 4:3 FTCA from 5:3 FTCA (Table 2). Although *Gordonia* sp. NB4‑1Y was previously observed to produce 5:3, 4:3, and 3:3 FTCAs from 6:2 FTSA or 6:2 FTAB when these served as organic sulfur sources; it showed no detectable transformation when supplied directly with 5:3 FTCA under our conditions (with inorganic sulfur available). The alkaline consortium (pH ≈ 10.3) produced a transient α/β‑hydroxy‑5:3 FTCA, indicating that hydroxylation can occur but did not progress to downstream chain‑shortening products. Further resting‑cell assays showed a similar pattern. Although our calculations indicate that defluorination is more favorable when the released H⁺ is efficiently neutralized/removed (often represented as balancing with OH⁻), this does not mean that simply raising the bulk medium pH will enhance defluorination in vivo. Microorganisms regulate intracellular pH near neutrality, so external alkalinity may not translate into improved proton management at the site of the enzymatic reaction. Conversely, organisms with strong proton‑handling capacity (e.g., efficient H⁺ export and tight control of proton gradients) could, in principle, better accommodate proton‑producing steps, regardless of whether they are alkaliphiles or acidophiles. Our observation that the alkalinophilic consortium accumulated only the hydroxylated intermediate supports the view that bulk alkalinity alone is insufficient. Future enrichments should therefore prioritize microbes and communities with effective proton and redox balancing, rather than screening only under alkaline conditions.

Overall, 5:3 FTCA transformation appears largely co-metabolic and community-dependent. It did not support growth or transformation in pure culture but was observed in mixed consortia given an auxiliary carbon source (e.g., methanol). This is consistent with our thermodynamic results, which suggest the overall pathway can be feasible (especially under alkaline conditions) but includes an energetically uphill hydroxylation step that may require consortium-level redox support and rapid intermediate turnover. Despite its fatty-acid-like structure, 5:3 FTCA also showed no evidence of entering conventional β-oxidation pathways. Its defluorination also appears mechanistically distinct from mono-fluorinated or chlorinated organics^25^.

## Discussion

The results above showed that standard isolation and enrichment strategies are ill-suited for identifying n:3 FTCA degraders. Conventional enrichment relies on selective media in which only organisms that use the target compound as a sole nutrient source (C/N/S) grow, or on selection via toxicity (e.g., antibiotics; fluoroacetate, tolerated/metabolized by a few specialists despite fluoride toxicity). Although 5:3 FTCA contains a non‑fluorinated alkyl segment that could, in principle, serve as a carbon source, our results indicate that n:3 FTCA neither serves as a growth substrate nor imposes measurable selective pressure. In co‑substrate tests, octanoic acid (an alkane analog) inhibited growth, consistent with reported membrane‑disruption antimicrobial effects^37,38^, whereas 5:3 FTCA did not cause comparable growth suppression in the tested microbial cultures. Because n:3 FTCAs are unlikely to support growth, any transformation is likely incidental or co‑metabolic, so degraders gain no advantage and are easily outcompeted or remain dormant. This makes consortium-based enrichment more realistic than isolating single strains, and overcoming the two bottleneck reactions may require syntrophic coupling, as in methanogenic degradation^30^, where an endergonic step can proceed when linked to a strongly exergonic partner reaction.

**Fig. 3:**
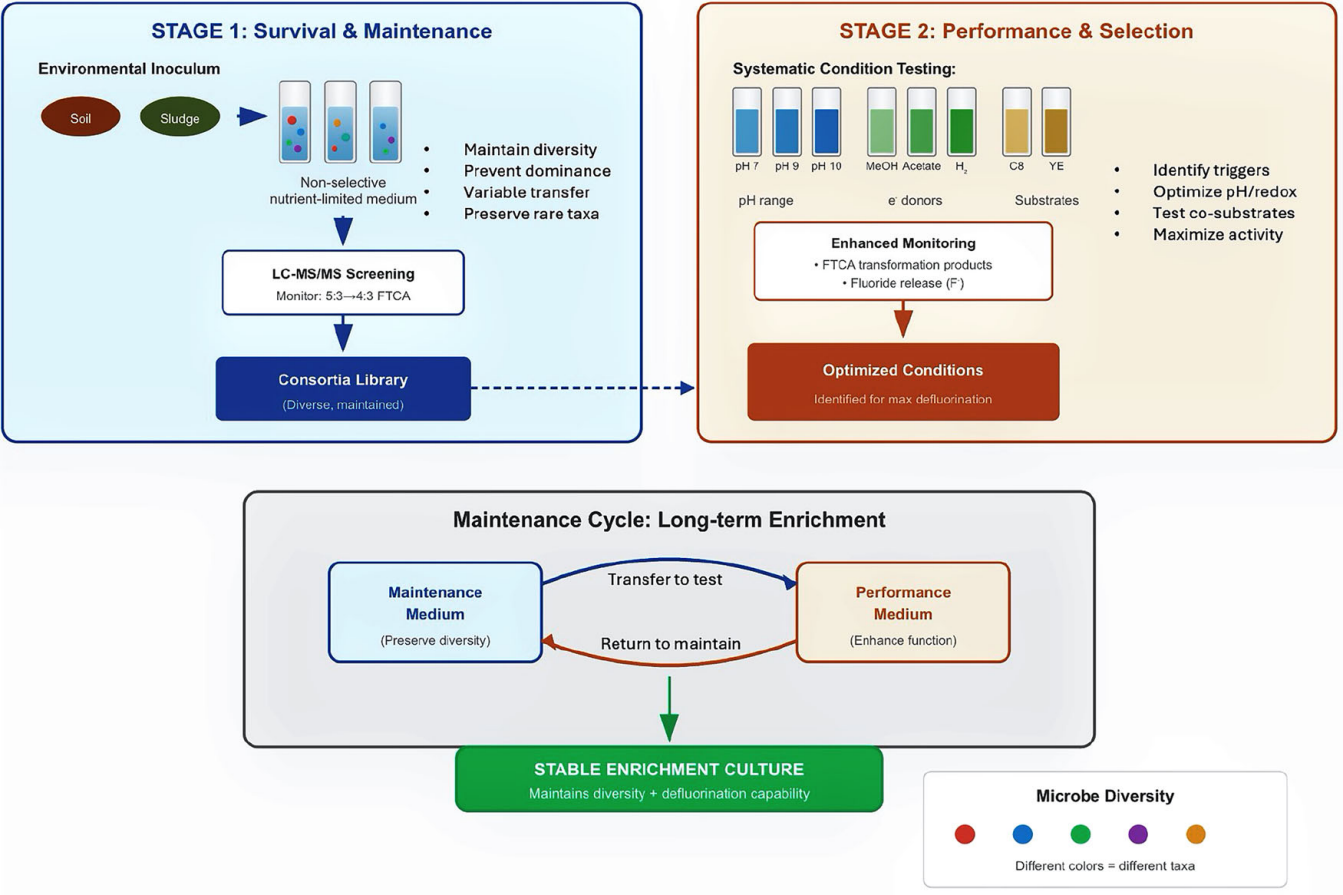
Proposed dual-stage enrichment strategy for cultivating n:3 FTCA-degrading microbial consortia. Stage 1 (Survival) maintains diverse consortia in non-selective, nutrient-limited media with periodic LC-MS/MS screening; Stage 2 (Performance) tests co-metabolic conditions (pH, electron donors, primary substrates) to identify triggers that enhance defluorination, with optional alternating transfers between two stages.

**Fig. 4:**
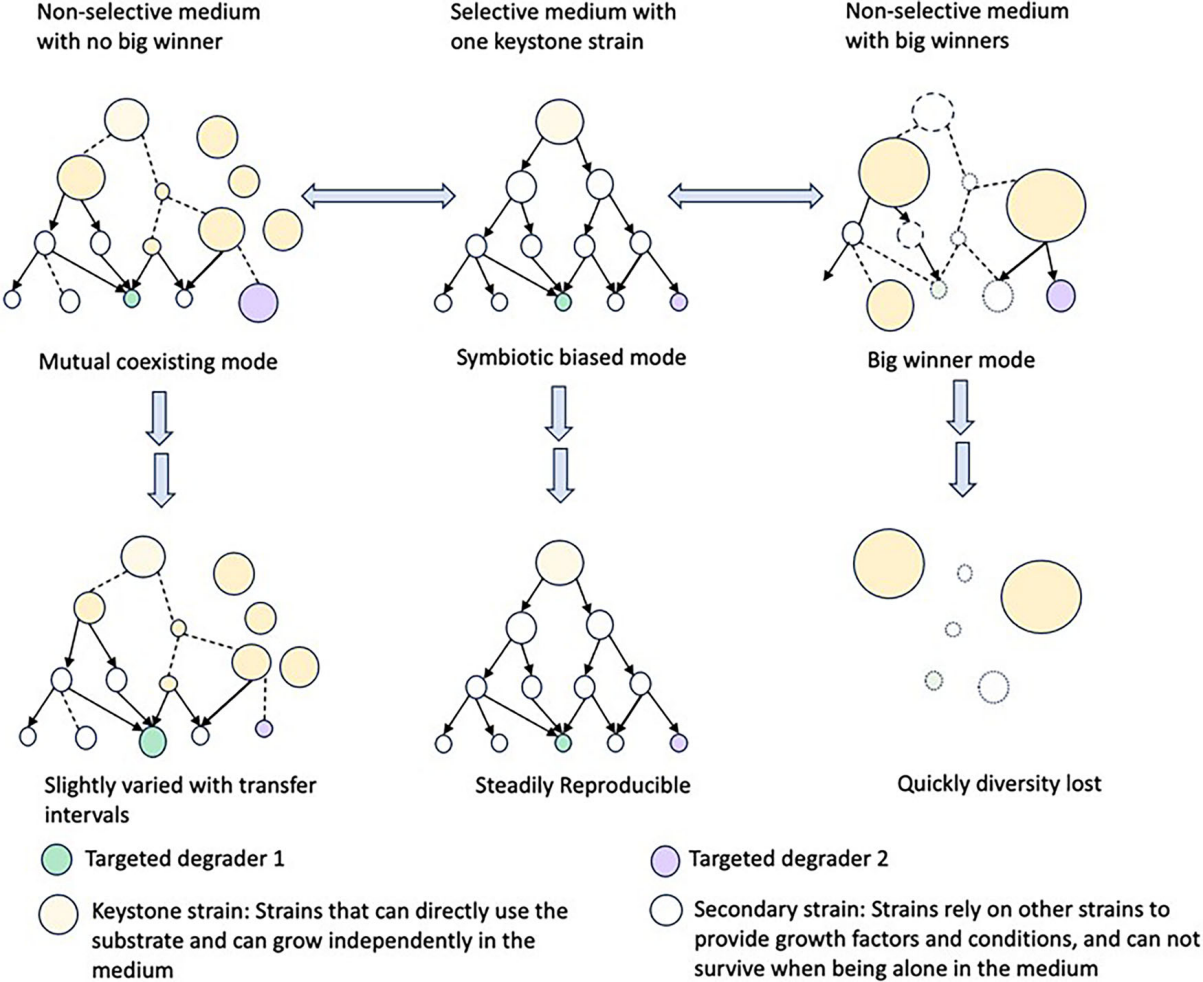
Schematics illustrating how culture media shape microbial consortia during enrichment. A typical consortium structure in a conventional selective medium (top middle) and two outcomes after transfer to a non-selective medium, either domination by a “big winner” (right) or mutual coexistence (left). The left strategy is proposed as most suitable for enriching 5:3-FTCA-degrading microbes.

Recognizing the absence of a natural selective force for 5:3 FTCA degradation via the one‑carbon removal pathway, we propose a dual‑stage enrichment strategy that separates degrader survival (Stage 1; Fig. 3) from degradative performance (Stage 2; Fig. 3). Stage 1 should prioritize survival and maintenance of potential degraders. A non-selective, nutrient-limited medium can be used to maintain community diversity and prevent domination by fast-growing organisms. Common substrates (e.g., glucose, yeast extract, and peptone) can be varied to generate a consortia library spanning “big-winner” and “coexisting” community modes (Fig. 4), and these consortia should be screened periodically for n:3 FTCA transformation. Because coexisting communities often produce low biomass, transformation should be assessed using high-sensitivity LC-MS/MS rather than growth curves alone. Early transfers typically preserve fast growers, whereas late transfers increase the risk of degrader starvation or washout. Variable transfer intervals in batch cultures or gradual nutrient re-addition in continuous bioreactors can be used to identify transfer conditions that maintain both diversity and degradative potential. Once a stable consortium is established, Stage 2 can focus on enhancing n:3 FTCA degradation and defluorination by systematically varying potential co‑metabolic triggers. These triggers can include the primary substrate, pH, and trace nutrients or electron donors. When a performance medium is identified, routine alternation between a Stage 1 maintenance medium and a Stage 2 performance medium can separate survival from function while retaining degradative capacity. Alternatively, a genuinely selective medium may be discovered that supports both consortium maintenance and defluorination in a single step.

This two‑stage framework also highlights community structure, especially stable coexistence, as a key design objective in Stage 1. Our experimental results support consortium-based enrichment over single-strain isolation, consistent with prior evidence that microbial communities are key functional units for xenobiotic transformation^39^. Because degradation was detected only after prolonged incubation, the responsible microbes are likely slow‑growing and dependent on partner metabolism. Thus, the main challenge at Stage 1 is to design a cultivation medium that preserves diversity while maintaining stable, transferable interspecies interactions. In conventional enrichment, a selective substrate can stabilize a consortium by supporting a keystone strain that feeds others via metabolite exchange (a “symbiotic‑biased mode,” Fig. 4, center). However, for 5:3 FTCA, an appropriate keystone‑selecting substrate is not apparent. Conversely, nutrient‑rich non‑selective media (e.g., nutrient broth) typically favor fast growers, leading to rapid diversity loss across transfers and the loss of slow‑growing specialists.

Thus, we propose a third enrichment scenario, the “mutual coexisting mode” (Fig. 4, left), in which community members persist across transfers without a single fast‑growing “winner” dominating, thereby maintaining high diversity through serial passages. Because microbes that coexist in nature may fail to coexist in the lab without sufficient niche diversity, the enrichment medium must support coexistence. Chemical‑mediated interactions can enable coexistence under laboratory conditions^40^, and community-level syntrophic interactions often emerge in multi‑species communities rather than stable pairs (e.g., methanogenic degradation)^30,41^. In practice, this mode benefits directly from maintaining high diversity. As illustrated in Fig. 4, this mode can persist over multiple generations and may even sustain greater diversity than selective enrichments, as additional strains can emerge as keystone species without strict interspecies dependencies. Strains that remain dormant under selective conditions may also become metabolically active in non‑selective environments. Among the three enrichment modes (Fig. 4), mutual coexistence preserves the greatest diversity and requires no highly specific selective force, making it the most promising approach for n:3 FTCA enrichment. A practical way to achieve this is by using a diluted, nutrient-restricted general-purpose medium that limits the growth of fast-growing species. This “slowed competition” allows balanced community development, though nutrient limitation alone cannot fully prevent dominance. Over time, competitive dynamics such as antibiotic production or toxin secretion may still disrupt balance, requiring ongoing monitoring and adjustment to sustain coexistence.

Collectively, the results suggest that fluorotelomer transformation is controlled less by intrinsic C-F bond strength alone and more by geochemical context and community metabolism. Thermodynamically, defluorination steps can be feasible, and overall favourability increases when protons are buffered or consumed, implying that buffered microenvironments and appropriate redox coupling can enable activity without requiring uniformly high bulk pH. Biologically, the pathway appears constrained primarily by upstream, energy‑demanding redox steps (notably dehydrogenation and hydroxylation) rather than by defluorination itself. This is consistent with our screening results, in which transformation was rare and low-yielding, observed only in methanol‑amended consortia that included methylotrophs. The environmental implication is that extensive fluorotelomer biotransformation is often slow, spatially heterogeneous, and consortium‑dependent, and may be most evident in systems that sustain diverse communities and syntrophic interactions (e.g., engineered reactors with long biomass retention). For engineered treatment, the most practical strategy is therefore to support sustained community‑level co‑metabolism, rather than to search for a single “PFAS degrader.” Because activity appears to reside in rare, slow‑growing consortia, treatment systems should prioritize high biomass retention and long contact times (e.g., biofilms, granules, membrane bioreactors, or activated sludge operated at long solid retention time) and consider auxiliary substrates that maintain supporting community functions.

Overall, the study contributes to evaluating the biodegradability of 5:3 FTCA by (1) providing quantitative thermodynamic evidence that HF‑elimination (defluorination) is not necessarily the dominant thermodynamic bottleneck in the one‑carbon removal pathway, (2) showing that n:3 FTCA transformation capacity appears to be rare, and (3) proposing an enrichment framework that separates microbial maintenance from functional selection.

## Methods

### Chemicals and reagents

5:3 FTCA (2H,2H,3H,3H-Perfluorooctanoic acid, 97% purity) and 4:3 FTCA (2H,2H,3H,3H-Perfluoroheptanoic acid, 97% purity) were purchased from SynQuest Laboratories (Alachua, FL, USA) and were used as the parent compounds for biotransformation experiments. Standards for other PFAS analytes and transformation products, including isotope‑labeled internal standards, were obtained from Wellington Laboratories (Guelph, ON, Canada). HPLC-grade methanol was purchased from Thermo Fisher (Mississauga, ON). Resazurin sodium salt was purchased from Sigma-Aldrich (St. Louis, MO, USA). Octanoic acid (99% purity) and citric acid (>99% purity) were purchased from Fisher Scientific (Canada). Deionized water (DI) was used throughout the study. All other reagents were commercial products of the highest grade available. The full list of PFAS standards is provided in Supplementary Table S1 of the Supplementary Materials.

Corning Costar 96-well flat-bottom tissue culture plates were purchased from Thermo Fisher. Culture media included Luria-Bertani (LB), Reasoner’s 2A (R2A), and Yeast extract (YE), all obtained from BD Difco^TM^. The mineral salts medium (MSM) contained the following components (per L): K₂HPO₄ 1.5 g, KH₂PO₄ 0.5 g, (NH₄)₂SO₄ 0.5 g, NH₄NO₃ 1 g, MgSO₄·7H₂O 0.2 g, and NaCl 1 g. The medium was supplemented with a trace element (TE) solution prepared as follows (per L): CaCl₂·2H₂O 3 g, FeSO₄·7H₂O 4.5 g, MnCl₂·4H₂O 0.99 g, ZnCl₂ 0.664 g, CoCl₂·6H₂O 0.24 g, Na₂MoO₄·2H₂O 0.24 g, H₃BO₃ 0.06 g, KI 0.17 g, CuCl₂·2H₂O 0.17 g, NiCl₂·6H₂O 0.186 g, and HNO_3_ (~50 mL) to aid dissolution. A yeast extract stock solution was prepared in DI water at 5% (w/v) for use as the vitamin source. All reagents were dissolved in DI water with continuous stirring until fully dissolved. After autoclaving, 4 mL of the TE solution and 8 mL of yeast extract stock solution (final concentration 0.04%) were added to 1 L of MSM to form the complete medium. During screenings that used a co-substrate, 79 µL of octanoic acid was added per L of MSM. Resazurin was prepared as a 10 mg/mL stock solution and used at a final concentration of 0.01 mg/mL in culture media. Solid media were prepared by adding 1.5% (wt/vol) agar.

### High-throughput screening of environmental microbes

#### Source of environmental microbes

Microbial communities from municipal activated sludge and PFAS‑contaminated soils were screened using a microplate-based dilution-to-extinction approach. Activated sludge was collected from a local wastewater treatment plant (Régie d’assainissement des eaux du bassin de Laprairie, Quebec, Canada). Three soils (designated Dorval, CAN, and Mirabel), selected based on the “OECD Guideline 304A-Inherent Biodegradability in Soil” and previously used in PFAS biotransformation studies (18), were also included. Sludge samples were mixed with MSM medium (1:5 m/v) and precultured overnight before library construction. Each soil sample was mixed with MSM medium (1:5 m/v), spiked with 10 ppm 5:3 FTCA, and amended with and without octanoic acid for four sequential passages. Octanoic acid, an eight-carbon analog structurally similar to 5:3 FTCA, was included to potentially stimulate microbial enzyme systems that might also act on the fluorinated compound. The PFAS profile of the four passages was determined before library construction (Supplementary Table S5).

#### Library construction and microplate assay

**Fig. 5:**
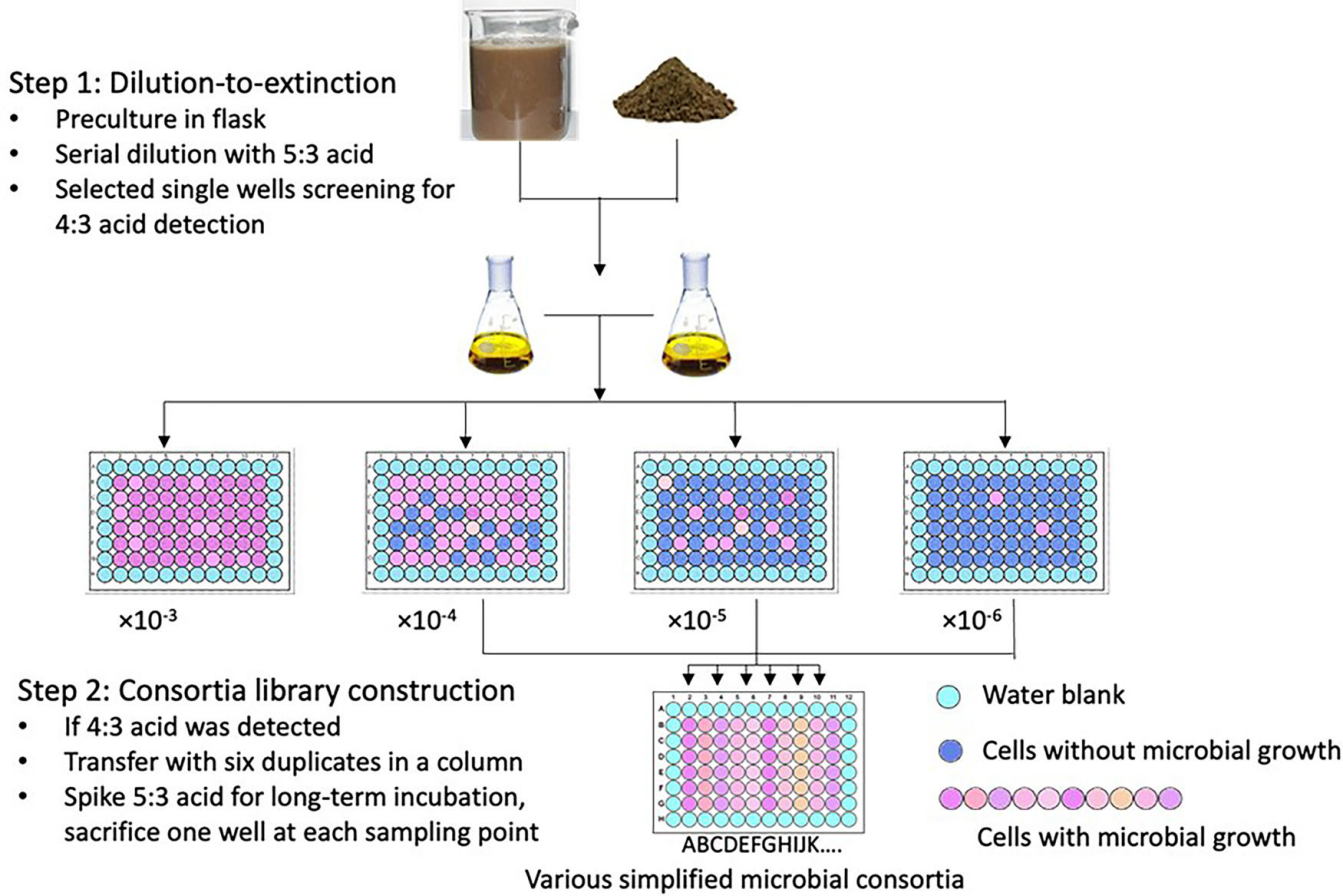
Workflow for consortium high-throughput screening with the dilution-to-extinction method. Dilution-to-extinction screening strategy used to identify metabolically active consortia associated with 5:3 FTCA biotransformation. Wells with a resazurin colour change were analyzed for 4:3 FTCA formation by LC-MS/MS and transferred to establish a screening library. Blue indicates no detectable metabolic activity, pink indicates metabolically active wells, and arrows indicate screening and transfer steps.

Enriched inocula from the above after four sequential passages were serially diluted in MSM medium (10^−3^ to 10^−6^) and inoculated into the 60 central wells of 96-well plates (Fig. 5, step 1). A 5 µL aliquot of 5:3 FTCA (dissolved in methanol) was added to each inner well, and the methanol was allowed to evaporate prior to inoculation. Each well was then filled with 200 µL of a diluted culture, resulting in a final 5:3 FTCA concentration of 10 ppm. The concentration of 10 ppm (~30 µM) 5:3 FTCA was selected based on: (1) prior biotransformation studies using similar concentrations^18^; (2) analytical detection limits requiring sufficient product formation for reliable quantification; and (3) the need to balance substrate availability with potential toxicity, as fluorotelomer acids exhibit antimicrobial effects at higher concentrations^42,43^. The outer wells were filled with sterile distilled water or MSM medium to create a moisture barrier and minimize evaporation from the inner wells. The plates were sealed with Parafilm and incubated at 24 °C in the dark.

#### Detection and transfer

After 7 days of incubation, wells showing a color change of the resazurin indicator from blue to pink at high dilution levels (10⁻⁴–10⁻⁶) were identified as containing viable microorganisms with detectable cellular redox activity. In this study, resazurin reduction was used only as a nonspecific indicator of cell viability and growth in highly diluted wells to prioritize wells for follow-up analysis. It was not used as evidence of PFAS transformation, defluorination, or one-carbon-pathway activity. These wells were then screened for 4:3 FTCA production from 5:3 FTCA by LC-MS/MS after an additional 7 days of incubation to allow detection of slower-growing microorganisms. The wells testing positive for 4:3 acid formation were transferred to new 96‑well plates in six replicates by mixing 150 µL of the culture with 1350 µL of fresh medium, with 200 µL of the mixture dispensed into each well. Plates containing these selected wells were designated as a microbial screening library (Fig. 5, step 2). During subsequent sampling events, one replicate well was sacrificed for PFAS analysis, and cultures with consistent degradation profiles were selected for further isolation and purification. All tests, including the ones described in the later sections, were performed at room temperature (25 °C), unless otherwise stated. Incubation periods of 7–14 days were selected to allow detection of slow-growing microorganisms while maintaining practical throughput for screening hundreds of wells. As discussed later, the extended observation period (~3 months at 4 °C) that revealed biotransformation in certain wells was an unplanned finding that underscores the importance of prolonged incubation for kinetically constrained degraders. This observation informed our proposal for extended enrichment strategies in the Discussion.

### Stain isolation and characterization

Microorganisms from positive wells that exhibited 5:3 FTCA biotransformation were isolated on R2A and LB agar plates via serial dilution. Colonies were purified through repeated streaking. Seven isolates were obtained and their identities were determined by PCR amplification and Sanger sequencing of the 16S rRNA gene using F1 (5’-GAGTTTQATCCTGGCTCAG-3’) and R13 (5’-AGAAAGGAGGTGATCCAGCC-3’) universal primers. The 25 μL reaction mixture contained 0.5 μM primers, 2 U Taq polymerase, 0.2 mM dNTP, and 1 μL DNA template. Sequencing was performed by La plateforme d’analyze génomique (PAG) de l’Université Laval (Canada), and sequence identity was confirmed by BLASTn searches against the NCBI nucleotide sequence database. Although these isolates were obtained from wells showing degradation activity, their individual ability to biotransform 5:3 FTCA was subsequently assessed, as described below.

### Screening of pure cultures and defined consortia

#### Pure strains

Four previously reported pure cultures, including *Gordonia* sp. NB4-1Y^27^, *Rhodococcus* sp. JVH1^28^, *Mycobacterium austroafricanum* IFP 2012^35^, and *Pseudomonas oleovorans*^36^ were tested for 5:3 FTCA biotransformation and defluorination. The first two strains were provided by Dr. Jonathan Van Hamme (Thompson Rivers University, Canada), and the other two were from the culture collection at the National Research Council of Canada (Montreal). Each strain was tested in its respective culture medium, as reported in the literature, and is listed in Table 2. Seven bacterial isolates (listed in Table 2) obtained from the enrichment sludge during the environmental microbe screening (Section “High-throughput screening of environmental microbes”) were also tested individually for their ability to degrade 5:3 FTCA. All tests were performed in batch incubations using modified MSM or R2A medium under aerobic conditions, in Erlenmeyer flasks sealed with cotton plugs to allow passive aeration. Cultures were incubated in the dark at 25 °C with shaking at 140 rpm. 5:3 FTCA (10 ppm) was added at the start of incubation, and the methanol solvent was evaporated before the culture medium was added. Each isolate was monitored for 14 days to evaluate the degradation of 5:3 FTCA.

#### Microbial consortia

Two pH-tolerant consortia from our lab were assessed for 5:3 FTCA biotransformation. An acid rock drainage consortium (≈80% *Acidithiobacillus* spp.) was tested in 10 mL of 9 K medium containing 44.2 g/L FeSO_4_·7H_2_O, pH 2, and incubated in 20-mL sealed glass vials under a N_2_ atmosphere, excluding oxygen. The experiment aimed to assess whether iron‑reducing metabolism could facilitate the transformation of 5:3 acid under these acidic, anoxic conditions. In the meantime, an alkaline consortium (*Brevundimonas diminuta* ≈ 55%; *Achromobacter* spp. ≈ 34%) was tested in a basal salt medium (pH ≈ 10.3) supplemented with 12.5 mM glycine, 5 mM L-methionine, and 20 mM L-glutamic acid, in 250-mL flasks with passive aeration from ambient air through a cotton plug, shaken at 140 rpm. Similar to other tests, 5:3 FTCA was added at a concentration of 10 ppm before the incubation tests.

#### Resting cell assays

To evaluate non-growth-linked biotransformation, the seven isolates described in Supplementary Table S9 and one representative consortium from which those isolates were obtained from the environmental screening study were grown in 100 mL R2A medium (48 h), harvested by centrifugation, washed, and resuspended in 20 mL MSM without added carbon. Cultures were spiked with 5:3 FTCA and incubated as described above. Periodically collected aliquots were analyzed for PFAS following the LC‑MS protocol described as below.

### PFAS quantification by LC-MS/MS and fluoride measurement

To monitor PFAS biotransformation products, 100 µL of inoculum was mixed with 900 µL of methanol and centrifuged at 10,000 × *g* for 10 min. A 190 µL aliquot of the supernatant was then spiked with 10 µL of internal standard solution containing mass-labeled [M + 4] PFBA and [M + 2] PFHxA to yield a final concentration of 2 ng mL⁻¹, followed by thorough mixing prior to quantification. PFAS analysis was performed using an AB Sciex QTRAP 5500 coupled to a Shimadzu HPLC system operated in negative electrospray ionization mode with multiple-reaction monitoring (see Supplementary Table S4 for monitored transitions). Separation was achieved on a Thermo Hypersil GOLD column (1.9 µm, 100 × 2.1 mm). The mobile phases consisted of 0.15% acetic acid in water (A) and 0.15% acetic acid in acetonitrile (B), run in gradient mode at 0.4 mL min⁻¹. The injection volume was 5 µL. Detailed LC–MS/MS parameters and detection limits are provided in Supplementary Table S4. Fluoride concentrations were measured using a combined ion-selective electrode (perfection comb F; Mettler Toledo, Canada). For the screening assay in Section “Screening of additional pure cultures and defined consortia”, 1.4 mL of culture was aseptically withdrawn, centrifuged at 10,000 × *g* for 10 min, and 1 mL of supernatant was mixed with an equal volume of TISAB II buffer in 20 mL HDPE vials before measurement. Calibration standards were prepared using NaF in the corresponding culture medium, and the method detection limit was 2 µM F⁻.

### Thermodynamics modeling method

#### Computational methodology and calculation guidelines

In parallel with the experimental screening, we performed DFT-based quantum chemical calculations to estimate the changes in Gibbs free energy of anionic PFAS and related reactants in water. The integration of findings from both computational and experimental components can provide a more complete basis for interpreting experimental observations and guiding future studies. All computations were conducted using the Gaussian^®^ 16 Revs. C.01 suit at the B3LYP/6‑311++G(2d,2p) level of theory with the SMD solvation model. The thermodynamic calculations aimed to evaluate the feasibility of each sub-step in the one-carbon removal pathway and to identify the conditions that could favor these reactions. The one-carbon removal pathway proposed in a 2012 study was used as the basis for these calculations^18^. For the PFCA formation pathway, certain sub-steps involving volatile intermediates were combined, and calculations focused on the key chemical intermediates as shown in Fig. 1. Free energy data for each compound under standard conditions were obtained from their optimized structures following frequency calculations; the values are provided in Supplementary Table S2, and the corresponding balanced reaction equations are listed in Supplementary Table S3. Each reaction was balanced under weakly acidic and weakly alkaline conditions, considering only O_2_, H_2_O, H^+^, OH^−^, and NADH/NAD^+^, where applicable. It is important to note that the Gibbs free energy changes (Δ*G*°) reported here were calculated under standard conditions (1 M reactants and products, 1 atm, 298 K), which do not necessarily reflect actual environmental or engineered systems. In real systems, the actual Gibbs free energy (Δ*G*) depends on the activities of the participating species, following the relationship Δ*G* = Δ*G*°+*RT* ln *Q*. Because PFAS and their intermediates typically occur at much lower concentrations in environmental matrices, the true Δ*G* values may deviate substantially from these standard estimates. Nonetheless, Δ*G*° values provide a consistent thermodynamic benchmark for evaluating more realistic environmental conditions.

#### Selection of compounds and establishing the pathway equations

The one-carbon removal pathway proposed by Wang et al., 2012 based on experimental observations can be divided broadly into three parts as shown in Fig. 1: (i) dehydrogenation and hydroxylation of n:3 acid to form α-OH-n:3 FTCA; (ii) decarboxylation of α-OH-n:3 FTCA to form n:2 FTCA; (iii) HF elimination of n:2 FTCA to form n:2 FTUCA, followed by reductive defluorination of n:2 FTUCA to form (n-1):3 FTCA. N:2 FTUCA can alternatively be defluorinated via the PFCA formation pathway, producing the volatile compound n:2 fluorotelomer ketone (n:2 ketone) and the secondary fluorotelomer alcohol (sFTOH), and finally forming PFCAs. Based on the three steps described above, we identified that the n:2 FTUCA represents a key branching point, where the substrates proceed through either the one-carbon removal pathway or the PFCA formation pathway. Therefore, the thermodynamic calculations were centered on this compound, with compounds from both pathways modeled. After the sums of electronic and thermal free energies for each compound were obtained, Δ*G*° values for each reaction were determined by taking the difference between the total free energies of products and reactants.

## Supplementary information

Supplementary Information

## Data Availability

The majority of the data supporting the findings of this study are included in this published article. The complete raw datasets generated and analyzed study are not publicly available due to their large file size but are available from the corresponding author upon reasonable request.
