# Supplementary material for: Enriching microbes capable of fluorotelomer acid defluorination: thermodynamic constraints and experimental challenges
Source: NPJ Emerg Contam. 2026 Jun 1;2(1):18. doi: 10.1038/s44454-026-00034-4 (PMC13226065; doi:10.1038/s44454-026-00034-4)
Supplement: Supplementary file 1 — Supplementary Information [file 44454_2026_34_MOESM1_ESM.pdf]

SUPPLEMENTARY MATERIALS

for

Enriching Microbes Capable of Fluorotelomer Acid Defluorination: Thermodynamic Constraints and Experimental Challenges

Dan Wang[1], Bei Yan[1], Nancy N. Perreault[2*], Jinxia Liu[1,3*]

[1] Department of Civil Engineering, McGill University, Montreal, QC, Canada

[2] National Research Council Canada, Montreal, QC, Canada

[3] Department of Civil and Environmental Engineering, Hong Kong Polytechnic University, Hung Hom, Hong Kong SAR

*Corresponding author:

Jinxia Liu: jinxia.liu@mcgill.ca; jinxia.liu@polyu.edu.hk

Nancy Perreault: Nancy.Perreault@cnrc-nrc.gc.ca

Table S1. Listing of fluorinated compounds modelled or analyzed in this study and their analytical methods

Part I: Fluorinated compounds modelled and analyzed

Chemical	Abbreviation	Structure	Cas No.	Vendor	Analytical method
2H,2H,3H,3H-Perfluorooctanoic FTCA	5:3 FTCA	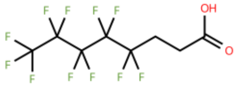	914637-49-3	SynQuest	HPLC-MS
2H,2H,3H,3H-Perfluoroheptanoic FTCA	4:3 FTCA	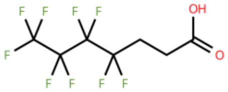	80705-13-1	SynQuest	HPLC-MS
2H,2H,3H,3H-Perfluorohexanoic FTCA	3:3 FTCA	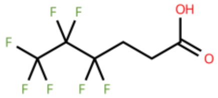	356-02-5	SynQuest	HPLC-MS
Perfluorohexanoic FTCA	PFHxA	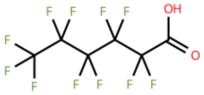	307-24-4	Wellington	HPLC-MS
Perfluoropentanoic FTCA	PFPeA	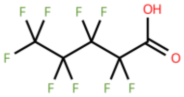	2706-90-3	Wellington	HPLC-MS
Perfluorobutanoic FTCA	PFBA	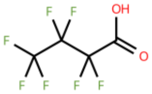	375-22-4	Wellington	HPLC-MS
α/β-hydroxy-2H,2H,3H,3H-Perfluorooctanoic FTCA	α/β-OH-5:3 FTCA	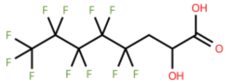 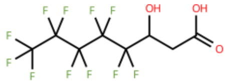	Not available	Not available, metabolite of microorganisms	LC-qTOF, HPLC-MS

Part II: Fluorinated compounds modelled but not analyzed

Chemical	Abbreviation	Structure	Cas No.
4,4,5,5,5-Pentafluoropentanoic FTCA	2:3 FTCA	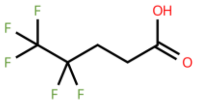	3637-31-8
4,4,4-Trifluorobutyric FTCA	1:3 FTCA	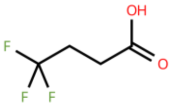	406-93-9
4,4,5,5,6,6,7,7,8,8,8-Undecafluoro-2-octenoic FTCA	5:3 FTUCA	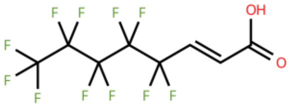	875878-70-9
4,4,5,5,6,6,7,7,7-Nonafluorohept-2-enoic FTCA	4:3 FTUCA	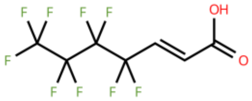	Not available

4,4,5,5,6,6,6-Heptafluoro-2-hexenoic FTCA	3:3 FTUCA	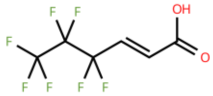	37759-76-5
4,4,5,5,5-Pentafluoropent-2-enoic FTCA	2:3 FTUCA	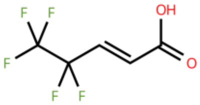	37759-75-4
4,4,4-Trifluorocrotonic FTCA	1:3 FTUCA	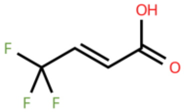	406-94-0
2H,2H-Perlfuorooctanoic FTCA	6:2 FTCA	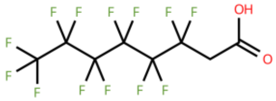	53826-12-3
3,3,4,4,5,5,6,6,7,7,7-Undecafluoroheptanoic FTCA	5:2 FTCA	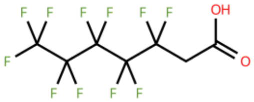	889944-77-8
3,3,4,4,5,5,6,6,6-Nonafluorohexanoic FTCA	4:2 FTCA	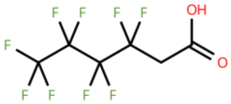	70887-89-7
3,3,4,4,5,5,5-Heptafluoropentanoic FTCA	3:2 FTCA	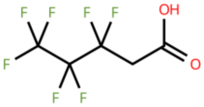	679-46-9
3,3,4,4,4-Pentafluorobutanoic FTCA	2:2 FTCA	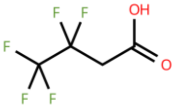	380-60-9
2H-Perfluoro-2-ocenoic FTCA	6:2 FTUCA	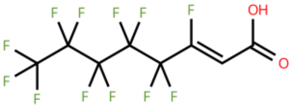	70887-88-6
3,4,4,5,5,6,6,7,7,7-Decafluorohept-2-enoic FTCA	5:2 FTUCA	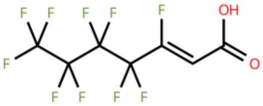	Not available
3,4,4,5,5,6,6,6-Octafluorohex-2-enoic FTCA	4:2 FTUCA	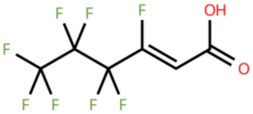	70887-90-0
3,4,4,5,5,5-Hexafluoropent-2-enoic FTCA	3:2 FTUCA	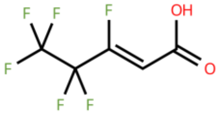	Not available
3,4,4,4-Tetrafluoro-2-butenoic FTCA	2:2 FTUCA	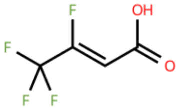	70887-91-1
α-hydroxy-2H,2H,3H,3H-Perfluorooctanoic FTCA	α-OH-5:3 FTCA	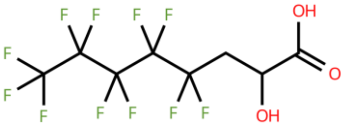	Not available

2-Hydroxy-4,4,5,5,6,6,7,7,7-nonafluoroheptanoic FTCA	α-OH-4:3 FTCA	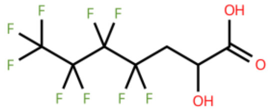	Not available
4,4,5,5,6,6,6-Heptafluoro-2-hydroxyhexanoic FTCA	α-OH-3:3 FTCA	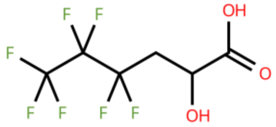	Not available
4,4,5,5,5-Pentafluoro-2-hydroxypentanoic FTCA	α-OH-2:3 FTCA	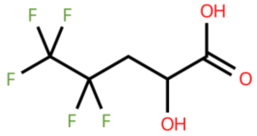	2228442-08-6
2,2,3,3,3-Pentafluoropropanoic FTCA	PFPrA	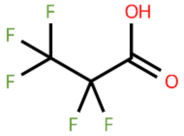	422-64-0
Trifluoroacetic FTCA	TFA	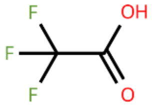	76-05-1

Table S2. Predicted sum of electronic and thermal free energies of each important compound in the pathway; all the calculations were performed at the B3LYP/6-311++G(2d,2p) level of theory using SMD as the solvation model (unit: Hartree)

n: 2 FTCAs/FTUCAs		n:3 FTCAs/FTUCAs		PFCAs and common reactants	
**6:2 FTCA**	-1755.123052	**5:3 FTCA**	-1556.576103	**PFHxA**	-1477.966875
**5:2 FTCA**	-1517.272354	**4:3 FTCA**	-1318.721667	**PFPeA**	-1240.114132
**4:2 FTCA**	-1279.419824	**3:3 FTCA**	-1080.871629	**PFBA**	-1002.261882
**3:2 FTCA**	-1041.568454	**2:3 FTCA**	-843.017637	**PFPrA**	-764.409182
**2:2 FTCA**	-803.716007	**1:3 FTCA**	-605.163379	**TFA**	-526.553072
**1:2 FTCA**	-565.861469	**5:3 FTUCA**	-1555.360974	H^+	-0.22518
**6:2 FTUCA**	-1654.629148	**4:3 FTUCA**	-1317.509349	H_2O	-76.471312
**5:2 FTUCA**	-1416.777309	**3:3 FTUCA**	-1079.656991	O_2	-150.328406
**4:2 FTUCA**	-1178.925591	**2:3 FTUCA**	-841.804055	OH^-	-75.987341
**3:2 FTUCA**	-941.072439	**1:3 FTUCA**	-603.948346	CO_2	-188.65607
**2:2 FTUCA**	-703.217819	**α-OH-5:3 FTCA**	-1631.823475	F^-	-100.043131
		**α-OH-4:3 FTCA**	-1393.971141	NAD^+	-2936.665477
		**α-OH-3:3 FTCA**	-1156.11907	NADH	-2937.393432
		**α-OH-2:3 FTCA**	-918.266733	Butyric FTCA	-307.298407
				Octanoic FTCA	-464.497094

Table S3. Predicted standard Gibbs free energy (ΔG^0) values and corresponding reaction equations for each step in the proposed degradation pathway under alkaline conditions; --all the calculations were performed at the B3LYP/6-311++G(2d,2p) level of theory using SMD as the solvation model.

**No.**	**Step in the pathway**	**Predicated $\Delta G°$ (kJ/mol)**	**Equation under alkaline condition**
**1**	6:2 FTUCA to PFHxA	-1365.67	6:2 $FTUCA^-$ + 2 O_2 + OH^- → $PFHxA^-$ + 2 CO_2 + F^- + H_2O
**2**	6:2 FTUCA to PFPeA	-1948.57	6:2 $FTUCA^-$ + 2.5 O_2 + 3OH^- → $PFPeA^-$ + 3 CO_2 + 3F^- + 2 H_2O
**3**	5:2 FTUCA to PFPeA	-1363.30	5:2 $FTUCA^-$ + 2 O_2 + OH^- → $PFPeA^-$ + 2 CO_2 + F^- + H_2O
**4**	5:2 FTUCA to PFBA	-1947.50	5:2 $FTUCA^-$ + 2.5 O_2 + 3OH^- → $PFBA^-$ + 3 CO_2 + 3 F^- + 2 H_2O
**5**	4:2 FTUCA to PFBA	-1361.90	4:2 $FTUCA^-$ + 2 O_2 + OH^- → $PFBA^-$ + 2 CO_2 + F^- + H_2O
**6**	4:2 FTUCA to PFPrA	-1944.92	4:2 $FTUCA^-$ + 2.5O_2 + 3OH^- → $PFPrA^-$ + 3 CO_2 + 3 F^- + 2 H_2O
**7**	3:2 FTUCA to PFPrA	-1363.09	3:2 $FTUCA^-$ + 2 O_2 + OH^- → $PFPrA^-$ + 2 CO_2 + F^- + H_2O
**8**	3:2 FTUCA to TFA	-1937.15	3:2 $FTUCA^-$ + 2.5 O_2 + 3OH^- → TFA^- + 3 CO_2 + 3F^- + 2 H_2O
**9**	2:2 FTUCA to TFA	-1358.81	2:2 $FTUCA^-$ + 2 O_2 + OH^- → TFA^- + 2 CO_2 + F^- + H_2O
**10**	6:2 FTUCA to 5:3 FTUCA	-123.40	6:2 $FTUCA^-$ + NADH → 5:3 $FTUCA^-$ + NAD^+ + F^-
**11**	5:2 FTUCA to 4:3 FTUCA	-123.97	5:2 $FTUCA^-$ + NADH → 4:3 $FTUCA^-$ + NAD^+ + F^-
**12**	4:2 FTUCA to 3:3 FTUCA	-122.29	4:2 $FTUCA^-$ + NADH → 3:3 $FTUCA^-$ + NAD^+ + F^-
**13**	3:2 FTUCA to 2:3 FTUCA	-122.85	3:2 $FTUCA^-$ + NADH → 2:3 $FTUCA^-$ + NAD^+ + F^-
**14**	2:2 FTUCA to 1:3 FTUCA	-119.99	2:2 $FTUCA^-$ + NADH → 1:3 $FTUCA^-$ + NAD^+ + F^-
**15**	5:3 FTCA to 5:3 FTUCA	-241.49	5:3 $FTCA^-$ + 0.5 O_2 → 5:3 $FTUCA^-$ + H_2O
**16**	4:3 FTCA to 4:3 FTUCA	-248.87	4:3 $FTCA^-$ + 0.5 O_2 → 4:3 $FTUCA^-$ + H_2O
**17**	3:3 FTCA to 3:3 FTUCA	-242.78	3:3 $FTCA^-$ + 0.5 O_2 → 3:3 $FTUCA^-$ + H_2O
**18**	2:3 FTCA to 2:3 FTUCA	-245.56	2:3 $FTCA^-$ + 0.5 O_2 → 2:3 $FTUCA^-$ + H_2O
**19**	1:3 FTCA to 1:3 FTUCA	-241.75	1:3 $FTCA^-$ + 0.5 O_2 → 1:3 $FTUCA^-$ + H_2O
**20**	5:3 FTCA to 5:3 FTUCA	+8.41	5:3 $FTCA^-$ + NAD+ + OH^- → 5:3 $FTUCA^-$ + NADH +H_2O
**21**	4:3 FTCA to 4:3 FTUCA	+1.03	4:3 $FTCA^-$ + NAD+ + OH^- → 4:3 $FTUCA^-$ + NADH +H_2O
**22**	3:3 FTCA to 3:3 FTUCA	+7.12	3:3 $FTCA^-$ + NAD+ + OH^- → 3:3 $FTUCA^-$ + NADH +H_2O
**23**	2:3 FTCA to 2:3 FTUCA	+4.35	2:3 $FTCA^-$ + NAD+ + OH^- → 2:3 $FTUCA^-$ + NADH +H_2O
**24**	1:3 FTCA to 1:3 FTUCA	+8.16	1:3 $FTCA^-$ + NAD+ + OH^- → 1:3 $FTUCA^-$ + NADH +H_2O
**25**	5:3 FTUCA to α-OH-5:3 FTCA	+23.13	5:3 $FTUCA^-$ + H_2O → α-OH-5:3 $FTCA^-$
**26**	4:3 FTUCA to α-OH-4:3 FTCA	+24.99	4:3 $FTUCA^-$ + H_2O → α-OH-4:3 $FTCA^-$
**27**	3:3 FTUCA to α-OH-3:3 FTCA	+24.24	3:3 $FTUCA^-$ + H_2O → α-OH-3:3 $FTCA^-$
**28**	2:3 FTUCA to α-OH-2:3 FTCA	+22.67	2:3 $FTUCA^-$ + H_2O → α-OH-2:3 $FTCA^-$

**29**	α-OH-5:3 FTCA to 5:2 FTCA	-650.74	α-OH-5:3 $FTCA^-$+ O_2 → 5:2 $FTCA^-$ + CO_2 + H_2O
**30**	α-OH-4:3 FTCA to 4:2 FTCA	-650.23	α-OH-4:3 $FTCA^-$+ O_2 → 4:2 $FTCA^-$ + CO_2 + H_2O
**31**	α-OH-3:3 FTCA to 3:2 FTCA	-652.07	α-OH-3:3 $FTCA^-$+ O_2 → 3:2 $FTCA^-$ + CO_2 + H_2O
**32**	α-OH-2:3 FTCA to 2:2 FTCA	-651.78	α-OH-2:3 $FTCA^-$+ O_2 → 2:2 $FTCA^-$ + CO_2 + H_2O
**33**	6:2 FTCA to 6:2 FTUCA	-87.16	6:2 $FTCA^-$ + OH^-→ 6:2 $FTUCA^-$ + F^- + H_2O
**34**	5:2 FTCA to 5:2 FTUCA	-84.17	5:2 $FTCA^-$ + OH^-→ 5:2 $FTUCA^-$ + F^- + H_2O
**35**	4:2 FTCA to 4:2 FTUCA	-86.30	4:2 $FTCA^-$ + OH^-→ 5:2 $FTUCA^-$ + F^- + H_2O
**36**	3:2 FTCA to 3:2 FTUCA	-81.62	3:2 $FTCA^-$ + OH^-→ 5:2 $FTUCA^-$ + F^- + H_2O
**37**	2:2 FTCA to 2:2 FTUCA	-75.91	2:2 $FTCA^-$ + OH^-→ 5:2 $FTUCA^-$ + F^- + H_2O
**38**	5:3 FTCA to 4:3 FTCA	-578.46	5:3 $FTCA^-$ + 0.5 O_2 + 2 OH^- → 4:3 $FTCA^-$ + H_2O + 2 F^- + CO_2
**39**	4:3 FTCA to 3:3 FTCA	-590.00	4:3 $FTCA^-$ + 0.5 O_2 + 2 OH^- → 3:3 $FTCA^-$ + H_2O + 2 F^- + CO_2
**40**	3:3 FTCA to 2:3 FTCA	-579.62	3:3 $FTCA^-$ + 0.5 O_2 + 2 OH^- → 2:3 $FTCA^-$ + H_2O + 2 F^- + CO_2
**41**	2:3 FTCA to 1:3 FTCA	-578.93	2:3 $FTCA^-$ + 0.5 O_2 + 2 OH^- → 1:3 $FTCA^-$ + H_2O + 2 F^- + CO_2
**42**	1:3 FTCA mineralization	-2310.77	1:3 $FTCA^-$ + 4 O_2 + 2 OH^- → 4CO_2+ 3 F^- + 2 H_2O
**43**	Octanoic acid to butyric acid	-3519.23	Octanoic $acid^-$ + 6 O_2 → Butyric $acid^-$ + 4 CO_2 + 4 H_2O
**44**	Octanoic acid mineralization	-6368.21	Octanoic $acid^-$ + 11 O_2 → 8 CO_2 + 7 H_2O + OH^-
**45**	Butyric acid mineralization	-2848.98	Butyric $acid^-$ + 5 O_2 → 4 CO_2 + 3 H_2O + OH^-
**46**	6:2 FTUCA to 5:2 FTUCA	-585.28	6:2 $FTUCA^-$ +0.5 O_2 +2 OH^-→ 5:2 $FTUCA^-$ +CO_2 +2 F^- + H_2O
**47**	5:2 FTUCA to 4:2 FTUCA	-585.59	5:2 $FTUCA^-$ +0.5 O_2 +2 OH^-→ 4:2 $FTUCA^-$ +CO_2 +2 F^- + H_2O
**48**	4:2 FTUCA to 3:2 FTUCA	-581.83	4:2 $FTUCA^-$ +0.5 O_2 +2 OH^-→ 3:2 $FTUCA^-$ +CO_2 +2 F^- + H_2O
**49**	3:2 FTUCA to 2:2 FTUCA	-577.97	3:2 $FTUCA^-$ +0.5 O_2 +2 OH^-→ 2:2 $FTUCA^-$ +CO_2 +2 F^- + H_2O
**50**	5:3 FTCA to 4:3 FTCA (acid condition)	+780.45	5:3 $FTCA^-$ + 0.5 O_2 + 2 OH^- → 4:3 $FTCA^-$ + H_2O + 2 F^- + CO_2
**51**	4:3 FTCA to 3:3 FTCA (acid condition)	+768.91	4:3 $FTCA^-$ + 0.5 O_2 + 2 OH^- → 3:3 $FTCA^-$ + H_2O + 2 F^- + CO_2
**52**	3:3 FTCA to 2:3 FTCA (acid condition)	+779.29	3:3 $FTCA^-$ + 0.5 O_2 + 2 OH^- → 2:3 $FTCA^-$ + H_2O + 2 F^- + CO_2
**53**	2:3 FTCA to 1:3 FTCA (acid condition)	+779.99	2:3 $FTCA^-$ + 0.5 O_2 + 2 OH^- → 1:3 $FTCA^-$ + H_2O + 2 F^- + CO_2
**54**	5:3 FTCA to PFPeA	-2316.57	5:3 $FTCA^-$ + 3.5 O_2 + 2 OH^- → $PFPeA^-$ + 3 H_2O + 2 F^- + 3 CO_2
**55**	5:3 FTCA to PFPeA (acid condition)	+224.76	5:3 $FTCA^-$ + 3.5 O_2 → $PFPeA^-$ + H_2O + 2 F^- + 3 CO_2 + 2 H^+
**56**	5:3 FTCA to PFBA	-2901	5:3 $FTCA^-$ + 4 O_2 + 4 OH^- → $PFBA^-$ + 4 H_2O + 4 F^- + 4 CO_2
**57**	5:3 FTCA to PFBA (acid condition)	-182.94	5:3 $FTCA^-$ + 4 O_2 → $PFBA^-$ + H_2O + 4 F^- + 4 CO_2 + 4 H^+
**58**	5:3 FTCA to PFBA (NAD^+)	-2650.86	5:3 $FTCA^-$ + 3.5 O_2 + 5 OH^- + NAD^+→ $PFBA^-$ + 4 H_2O + 4 F^- + 4 CO_2 + NADH

Table S4. Instrumental method parameters for quantitative analysis of 5:3 FTCA and transformation products by LC-MS/MS.

Instrument	Shimadzu High Performance Liquid Chromatograph with an AB Sciex Qtrap 5500 mass spectrometer. The mass spectrometer was operated in the negative ion multiple reaction-monitoring mode.				
Method Name in the software	5-3 FTCA biodegradation products				
Analytical Column	Thermo Hypersil GOLD (Part No. 25002-102130), 1.9 µm,100 mm x 2.1 mm				
Column Temperature	40ºC				
Mobile phases	A: 0.1% (v/v) acetic FTCA in LC-MS water<br>B: 0.1% (v/v) acetic FTCA in acetonitrile				
Gradient Profile	Time (min)			Percentage B	Flow Rate (mL/min)
	0.01			10	0.55
	5.00			72.5	0.55
	6.00			100	0.55
	10.80			100	0.55
	10.90			10	0.55
	11.00			Stop	
Injection Volume	5 µL				
Integrated Valco Valve	Position of Step 0: Waste (B)				
	Time (min)			Position	
	2.0			MS (A)	
	6.5			MS (A)	
	7.0			Waste (B)	
Monitored Ion Transitions	Analytes	LOD* µg L^{-1}	Linear Range	Retention Time (min)	Ion Transitions **(the most sensitive)**
	5:3 FTCA	0.5	0.5-100	5.10	341>237.0
					**341>217.0**
	4:3 FTCA	0.5	0.5-128	4.66	291>166.9
					**291>186.8**
	3:3 FTCA	2.5	2.5-50	4.14	**241>116.6**
					241>136.8
					241>157
	PFBA	0.1	0.1-50	2.49	213>169
	[M+4] PFBA				217>172
	PFPeA	0.1	0.1-25	3.43	263>219
	PFHxA	0.1	0.1-25	4.19	**313>269**
					313>119
	[M+2] PFHxA				315>270
	*LOD: Limit of detection defined as the lowest calibration standard in µg L^{-1}.				
LC/MS/MS Analog Parameters	Curtain Gas (CUR) 30<br>Collision Gas (CAD) 10<br>IonSpray Voltage (IS) -4500<br>Temperature (TEM): 550<br>Ion Source Gas 1 (GS1): 50<br>Ion Source Gas 2 (GS2): 50				

Table S5. PFAS profiles in pre-cultures of three soil samples (Dorval, CAN, Mirabel) incubated in 150 mL flasks for four successive passages prior to the dilution-to-extinction experiment (10-fold dilution; ng mL^{-1}). Octanoic FTCA (OA) was used as a carbon co-substrate.

Sample	PFPeA	PFHxA	PFHpA	PFBA	4:3 FTCA	5:3 FTCA
**Dorval 1**	31.8	34.6	1.34	2.28	6.64	1460
**Dorval 2**	5.49	14.6	n.d	n.d	0.888	873
**Dorval 3**	1.79	12.6	n.d	n.d	n.d	549
**Dorval 4**	1.42	13.2	n.d	n.d	n.d	641
**Dorval with OA 1**	27.9	26.3	1.05	1.48	3.53	1020
**Dorval with OA 2**	7.38	17.7	n.d	n.d	1.16	980
**Dorval with OA 3**	3.31	10.9	n.d	n.d	0.934	425
**Dorval with OA 4**	3.29	15.9	n.d	n.d	1.4	937
**CAN 1**	22.4	47.8	1.86	2.24	6.65	1110
**CAN 2**	5.78	21	n.d	n.d	2.4	1030
**CAN 3**	2.42	12.8	n.d	n.d	1.23	560
**CAN 4**	0.875	11.3	n.d	n.d	n.d	672
**CAN with OC 1**	11.8	50.2	2.08	2.17	2.31	1180
**CAN with OC 2**	5.73	25.6	n.d	n.d	1.69	1130
**CAN with OC 3**	1.48	11.5	n.d	n.d	n.d	486
**CAN with OC 4**	1.55	17.3	n.d	n.d	n.d	971
**Mirabel 2**	5.54	15.4	n.d	n.d	2.17	164
**Mirabel 3**	2.63	12.4	n.d	n.d	1.11	471
**Mirabel with OC 1**	22.4	25.6	n.d	n.d	1.82	1010
**Mirabel with OC 2**	6.12	17.8	n.d	n.d	1.39	953
**Mirabel with OC 3**	2.89	10.3	n.d	n.d	0.642	411
**Mirabel with OC 4**	1.94	11.4	n.d	n.d	0.515	444

For Table S5, three soil samples from the contaminated site were mixed with MSM at a 1:4 (m/v) ratio, and 50 mL of the resulting suspension was pre-cultured with 5:3 FTCA in 150 mL Erlenmeyer flasks. The cultures were transferred every seven days, with and without octanoic acid, for a total of four successive passages. Samples collected before each transfer were analyzed for PFAS transformation products. Preliminary analyses indicated that the presence of octanoic acid appeared to promote the formation of 4:3 FTCA, suggesting a potential co-metabolic effect. Based on these observations, the consortium-level screening of soil samples was subsequently conducted under both conditions (with and without octanoic acid) using 96-well microplates. However, during these assays, no biofilm formation or measurable 5:3 FTCA degradation occurred in the wells supplemented with octanoic acid. Likewise, no degradation activity was observed in the absence of octanoic acid, suggesting that under the tested conditions, direct metabolism or co-metabolic transformation of 5:3 FTCA was likely limited.

Table S6. Screening results from selected wells of sludge samples incubated in 96-well plates for biofilm, red flocs formation and the biodegradation confirmed by LC-MS/MS, showing the degradation correlated with the presence of biofilm and red flocs.

	Well ID	Biofilm	Red flocs	Biodegradation confirmed by LC-MS/MS
**1**	d53	Y	Y	Y
**2**	j515	Y	Y	Y
**3**	K2-515	Y	Y	Y
**4**	M-515	Y	Y	Y
**5**	10-4C-515	N	N	N
**6**	53A/B/C/D	Y	N	N
**7**	53E	Y	Y	Y
**8**	d53un	N	N	N
**9**	d515-4C	N	N	N
**10**	j53-un	Y	Y	Y
**11**	G53	Y	N (white dot)	Y
**12**	J626	N	N	N
**13**	K1-4C-515	N	N	N
**14**	K2-4C-515	Y	N	N
**15**	L-53-un	Y	N	N
**16**	M-53-un	Y	N	N
**17**	N-53-un	N	N	N
**18**	O-53-un	Y	N	N

Table S7. PFAS profiles from the 24-hour resting cell assay using the pink isolate (*Methylobacterium* sp.) and its source consortium (10-fold dilution; ng mL^{-1}). PFHxA was detected in the analytical control, originating as an impurity from the 5:3 FTCA standard.

	**Sample**	**5:3 FTCA**	**PFHxA**	**PFPeA**	**PFBA**	**4:3 FTCA**
**Consortium**	Medium control 24h	n.d	n.d	n.d	n.d	n.d
	Analytical control with 10 ppm 5:3 FTCA-24h	349	2.13	n.d	n.d	n.d
	Analytical control with 20 ppm 5:3 FTCA-24h	783	7.15	n.d	n.d	n.d
	Consortium 10 ppm-3h	525	4.48	n.d	n.d	n.d
	Consortium 10 ppm-24h	861	7.57	n.d	n.d	n.d
	Consortium 10 ppm-96h	914	9.45	n.d	1.4	n.d
	Consortium 20 ppm-3h	4200	57.7	3.13	3.4	n.d
	Consortium 20 ppm-24h	1600	19.6	1.17	0.734	n.d
	Consortium 20 ppm-96h	268	2.53	n.d	n.d	n.d
Methylobacterium sp.	Medium control-3h	n.d	n.d	n.d	n.d	n.d
	Medium control-24h	n.d	n.d	n.d	n.d	n.d
	Analytical control 10 ppm -24h	956	11.5	n.d	n.d	n.d
	Analytical control 20 ppm -24h	2070	15.9	n.d	n.d	n.d
	Pink 10 ppm-3h	603	4.65	n.d	0.11	n.d
	Pink 10 ppm-24h	841	7.06	n.d	n.d	n.d
	Pink 20 ppm-3h	2210	22.5	0.41	n.d	n.d
	Pink 20 ppm-24h	3050	34.9	1.99	n.d	n.d

Table S8. PFAS profiles from 14-day batch culture tests on 20 individual isolate candidates and their mixed cultures grown in R2A and other media (10-fold dilution; ng mL^{-1}).

Isolate	5:3 FTCA	PFHxA	PFPeA	PFBA	4:3 FTCA
F1	419	3.21	n.d	n.d	n.d
F2	540	4.89	n.d	n.d	n.d
F3	527	4.51	n.d	n.d	n.d
M1	510	6.19	n.d	n.d	n.d
T4	418	4.36	n.d	n.d	n.d
T1	661	6.48	n.d	n.d	n.d
Y1	438	4.68	n.d	n.d	n.d
Y3	350	3.54	n.d	n.d	n.d
Y2	465	5.79	n.d	n.d	n.d
F4	593	5.66	n.d	n.d	n.d
F5	423	4.42	n.d	n.d	n.d
F6	648	7.18	1.33	n.d	n.d
T3	590	5.96	n.d	n.d	n.d
L2	659	5.29	n.d	n.d	n.d
W1	613	4.9	0.903	n.d	n.d
S1	602	6.15	0.634	n.d	n.d
B1	301	5.49	0.737	n.d	n.d
S2	572	6.46	n.d	n.d	n.d
L1	398	5.26	n.d	n.d	n.d
L1YC	521	6.87	0.447	n.d	n.d
Mixed-LB	508	4.95	n.d	n.d	n.d
Mixed-MSM	546	6.3	n.d	n.d	n.d
Mixed-R2A	732	7.41	n.d	n.d	n.d

Table S9. Phylogenetic identities of seven morphologically distinct isolates based on BLASTn sequence analysis of partial 16s rRNA gene sequence (around 750 base pairs)

Strain ID	Taxonomy	Identities%	Other features
**R1**	*Methylobacterium extorquens*	98.2	Gram-negative
**Y1**	*Sphingomonas paucimobilis*	99.5	Gram-negative
**M1**	*Stenotrophomonas acidaminiphila*	98.3	Gram-negative
**F1**	*Bosea thiooxidans*	91.2	Gram-negative, sodium thiosulfate oxidizer
**F2**	*Mycolicibacterium cosmeticum*	94.0	actinobacteria
**W1**	*Thermomonas fusca*	98.3	Cyanide-degrading
**Y2**	*Bosea* sp.	98.9	nitrogen fixation

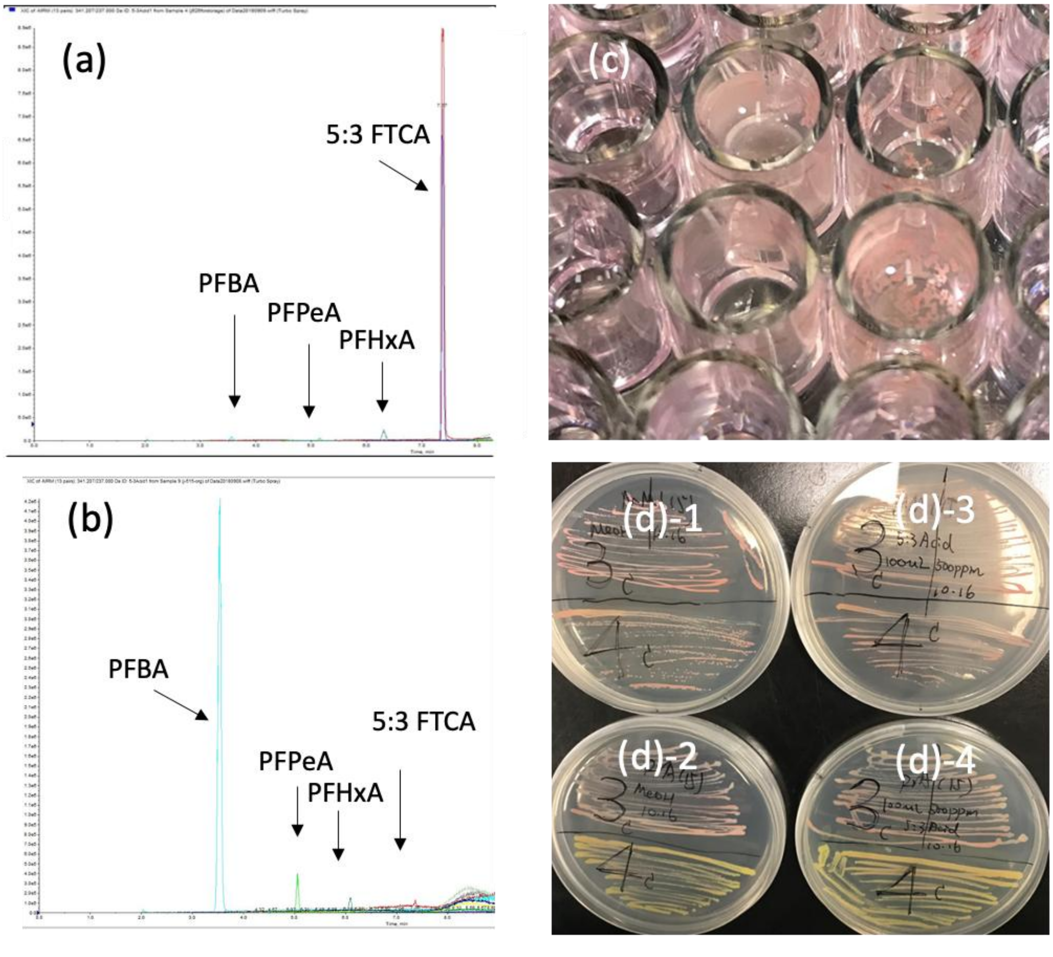

Fig. S1: Observed biotransformation of 5:3 FTCA in wells showing pink pigmentation and biofilm formation. (a) LC-MS profile from a well showing microbial activity but lacking biofilm after an additional 3 months of incubation at 4°C, which appeared similar to the analytical control. PFHxA was detected as an impurity in 5:3 FTCA, whereas PFBA and PFPeA were at or below the detection limits. (b) LC-MS profile from a well containing pink flocs and biofilm after 3 additional months of incubation at 4 ºC. (c) Photograph of a typical well displaying floating pink flocs on the surface. (d) Photographs showing pink flocs streaked onto MSM and R2A agar plates with or without 5:3 FTCA, respectively. Panels (d)-1 and (d)-3 correspond to MSM plates with and without 5:3 FTCA; panels (d)-2 and (d)-4 correspond to R2A plates with and without 5:3 FTCA.
